# GATA2 deficiency syndrome: A decade of discovery

**DOI:** 10.1002/humu.24271

**Published:** 2021-08-31

**Authors:** Claire C. Homan, Parvathy Venugopal, Peer Arts, Nur H. Shahrin, Simone Feurstein, Lesley Rawlings, David M. Lawrence, James Andrews, Sarah L. King‐Smith, Natasha L. Harvey, Anna L. Brown, Hamish S. Scott, Christopher N. Hahn

**Affiliations:** ^1^ Department of Genetics and Molecular Pathology SA Pathology Frome Road Adelaide South Australia 5000 Australia; ^2^ Molecular Pathology Research Laboratory, Centre for Cancer Biology SA Pathology and University of South Australia Adelaide South Australia 5000 Australia; ^3^ Section of Hematology/Oncology, Department of Medicine The University of Chicago Chicago Illinois USA; ^4^ Australian Cancer Research Foundation Cancer Genomics Facility Centre for Cancer Biology, SA Pathology Frome Road Adelaide South Australia 5000 Australia; ^5^ Specialist Genomics Australian Genomics 50 Flemington Road Parkville Victoria 3052 Australia; ^6^ Adelaide Medical School University of Adelaide Adelaide South Australia 5000 Australia; ^7^ Clinical Health Sciences University of South Australia Adelaide South Australia 5000 Australia

**Keywords:** GATA2 deficiency syndrome, germline variants, immunodeficiency, lymphedema, myeloid malignancy, predisposition

## Abstract

GATA2 deficiency syndrome (G2DS) is a rare autosomal dominant genetic disease predisposing to a range of symptoms, of which myeloid malignancy and immunodeficiency including recurrent infections are most common. In the last decade since it was first reported, there have been over 480 individuals identified carrying a pathogenic or likely pathogenic germline *GATA2* variant with symptoms of G2DS, with 240 of these confirmed to be familial and 24 de novo. For those that develop myeloid malignancy (75% of all carriers with G2DS disease symptoms), the median age of onset is 17 years (range 0–78 years) and myelodysplastic syndrome is the first diagnosis in 75% of these cases with acute myeloid leukemia in a further 9%. All variant types appear to predispose to myeloid malignancy and immunodeficiency. Apart from lymphedema in which haploinsufficiency seems necessary, the mutational requirements of the other less common G2DS phenotypes is still unclear. These predominantly loss‐of‐function variants impact GATA2 expression and function in numerous ways including perturbations to DNA binding, protein structure, protein:protein interactions, and gene transcription, splicing, and expression. In this review, we provide the first expert‐curated ACMG/AMP classification with codes of published variants compatible for use in clinical or diagnostic settings.

## Graphical abstract

GATA2 mutation update including a comprehensive deconvoluted summary of all published germline GATA2 deficiency syndrome mutations to date, associated phenotypes, and expert ACMG/AMP classification (with criteria) of all 179 pathogenic and likely pathogenic unique variants.

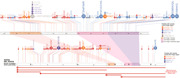

## 1 BACKGROUND

GATA2 deficiency syndrome (G2DS) (MIM#s 601626, 614286, 614038, 614172; GATA2 deficiency with susceptibility to MDS/AML, MONDO:0042982) is a collective of hematological (malignant and nonmalignant) and nonhematological phenotypes due to germline predisposing variants in the *GATA2* gene that act in a partially penetrant autosomal dominant manner. Symptoms may range from life‐threatening bone marrow (BM) failure, immunodeficiency, and/or myeloid malignancy, to no overt phenotype even at old age, although the latter is less common. Phenotypes that have been reported include myeloid malignancies (predominantly myelodysplastic syndrome [MDS] and acute myeloid leukemia [AML]), lymphedema (Emberger syndrome), and immune deficiency (DCML deficiency; combined deficit of DC, monocyte, B and NK lymphoid cells, MonoMAC; monocytopenia and mycobacterial infection) with associated recurrent infections (Dickinson et al., 2011; Donadieu et al., 2018; Emberger et al., 1979; Hahn et al., 2011; Hsu et al., 2011; Kazenwadel et al., 2012; Ostergaard et al., 2011; Spinner et al., 2014; Wlodarski et al., 2016). Other less common phenotypes include chronic neutropenia (Pasquet et al., 2013), cytopenia/BM failure (Ganapathi et al., 2015), pulmonary alveolar proteinosis (PAP), sensorineural deafness, neurological features, urogenital malformations (Donadieu et al., 2018; Spinner et al., 2014), thrombosis, autoimmune features, rheumatological features, premature labor, and miscarriage (Donadieu et al., 2018). Affected individuals may experience multiple phenotypes throughout their lifetime or even at a single time point, while others may present with only very mild symptoms.

To date, no single underlying common phenotype has been described in the majority of G2DS carriers, in contrast to platelet function disorder or thrombocytopenia in Familial platelet disorder with predisposition to acute myeloid leukemia (FPD/AML, MONDO:0011071) caused by germline pathogenic variants in the *RUNX1* gene. Therefore, it is proposed that each G2DS phenotype arises due to biological or environmental stressors that act on particular cell types in which reduced functional GATA2 protein is at or near a threshold level, thereby creating a situation in which GATA2 activity becomes limiting for normal cellular function. These “stressor events” may occur during embryogenesis or after birth and their effects accumulate over time. A mechanism has been proposed for “hematopoietic stem cell exhaustion” due to recurrent or persistent infections resulting in BM failure (Hirabayashi et al., 2017; Hsu et al., 2015). One might propose similar mechanisms for each of the phenotypes such as physical, inflammatory, or infectious stresses on lymphatic vessels during development causing lymphedema (Kazenwadel et al., 2015) or more stochastic events leading to malformations of the auditory system or urinary tract. For myeloid malignancies, germline *GATA2* variants may create a microenvironment that is conducive for the selection and clonal expansion of particular acquired mutations such as *ASXL1*, *CEBPA*, *SETBP1*, and −7/7q through a process coined “predestination” where a limited trajectory of disease evolution is imposed by pre‐existing variants (i.e., germline) or early (i.e., somatic) mutations (Papaemmanuil et al., 2013). Here we provide a comprehensive *GATA2* variant update of published germline cases and include unpublished variants from our institution cohort.

### 1.1 Ascertainment criteria of *GATA2* variants

**Figure 1:**
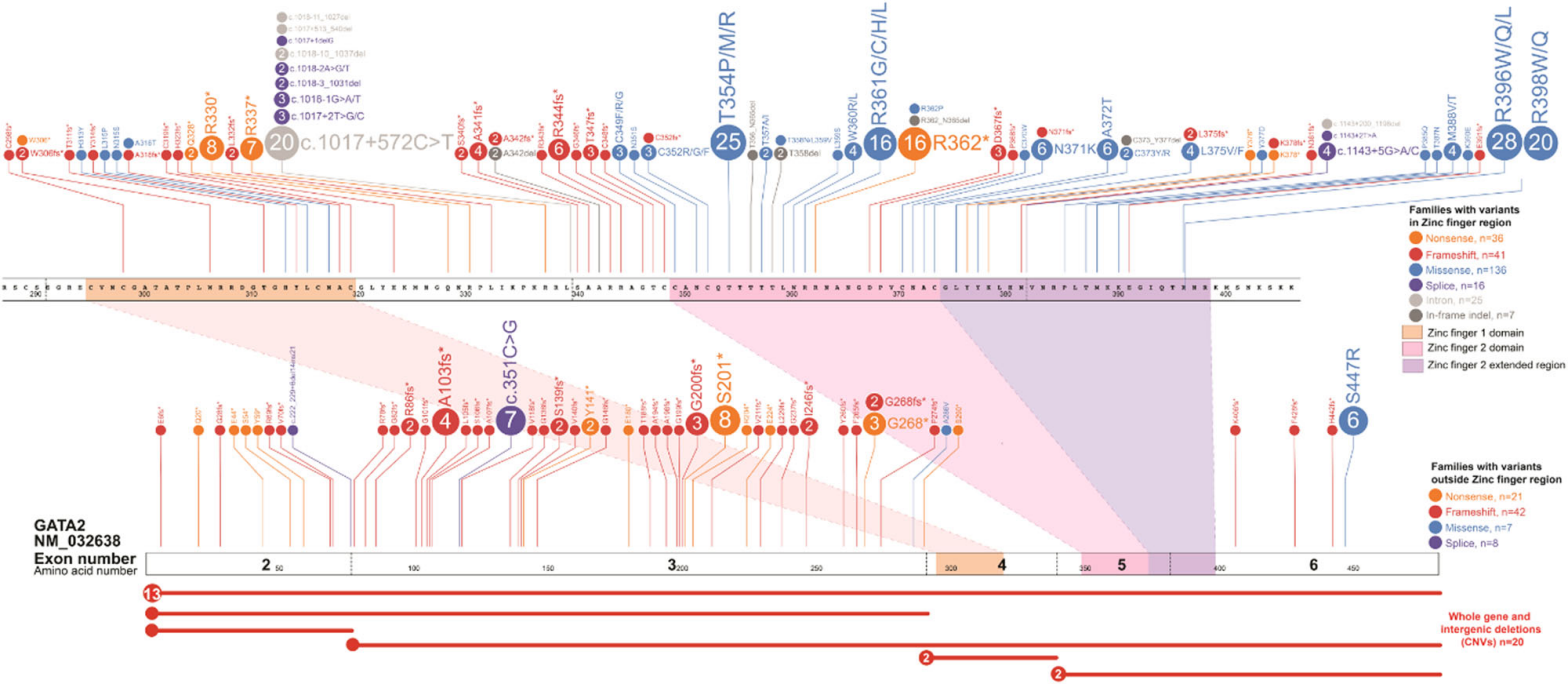
Germline GATA2 variants. All ascertained germline *GATA2* variants are visualized using the ProteinPaint web application (https://pecan.stjude.cloud/home) (Zhou et al., 2016). Variants (displayed as protein changes where possible) are color‐coded according to mutation effect. The number of probands for each variant is indicated within the circle where the number is greater than one. All variants are annotated to NM_032638.5

**Figure 2:**
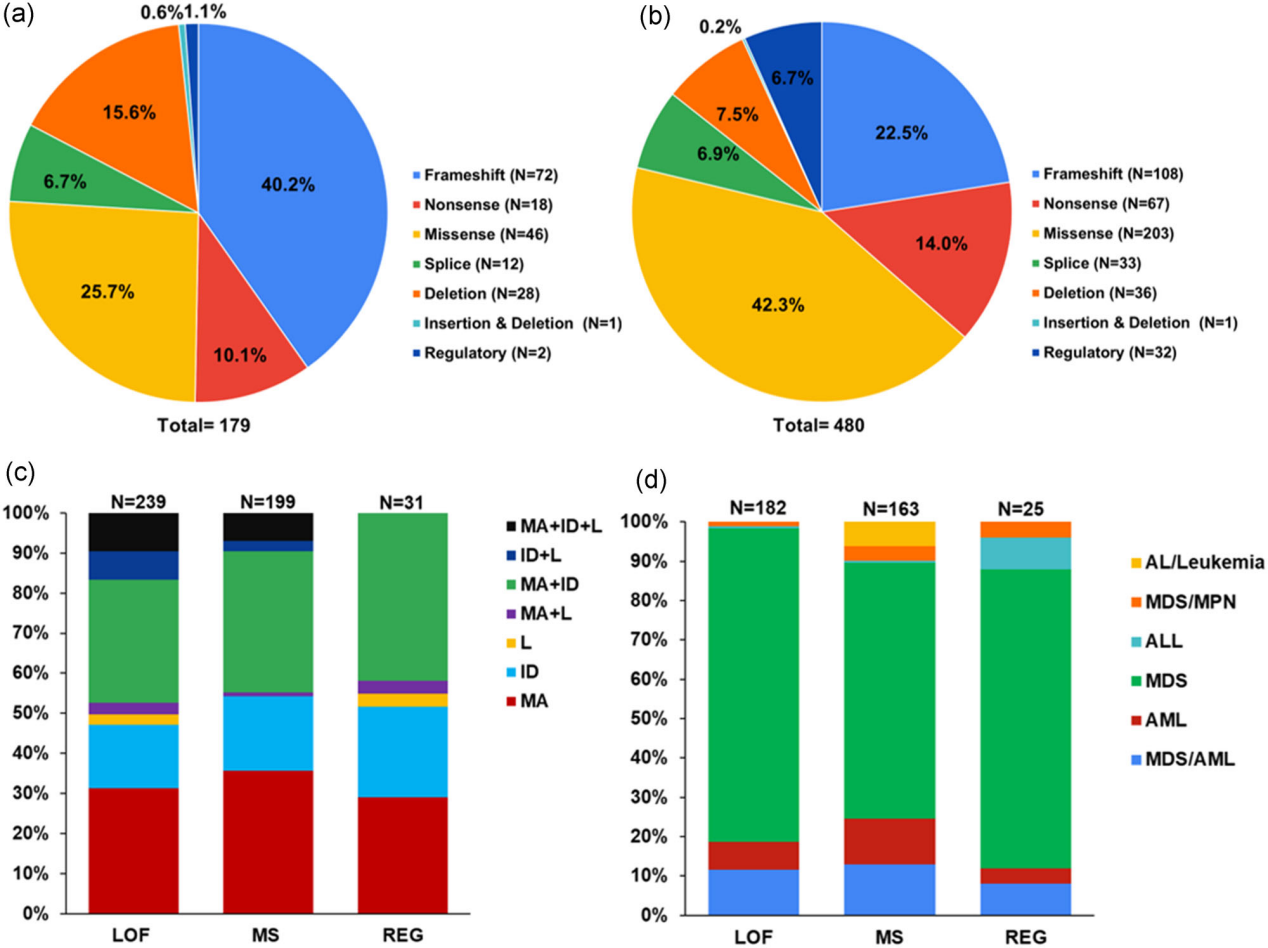
Prevalence of *GATA2* variant type and associated G2DS phenotypes. (a) Number and type of unique *GATA2* variants. (b) Total number and variant type of *GATA2* individuals. (c) Percentage of individuals with each *GATA2* variant type associated with each phenotype. (d) Percentage of individuals with each *GATA2* variant type with associated hematological malignancies. Loss‐of‐function (LOF), frameshift (FS), nonsense (NS), missense (MS), splice site (SPL), large deletion (DEL), small insertion/deletion (INDEL), regulatory (REG), all variants combined (total). Overall phenotypes: L, lymphedema; M/A, MDS and/or AML; ID, immunodeficiency; malignancy types: myelodysplastic syndrome (MDS), acute myeloid leukemia (AML), myeloproliferative neoplasm (MPN). B‐ or T‐cell acute lymphoblastic leukemia (ALL), acute leukemia (AL).

Familial and de novo germline *GATA2* variants were identified and extracted from peer‐reviewed literature and collated (Table 1 and Table S1). *GATA2* variants are described according to Human Genome Variation Society nomenclature and annotated to GenBank accession number NM_032638.5. Variants were classified according to American College of Medical Genetics and Genomics (ACMG)/Association for Molecular Pathology (AMP) guidelines (Richards et al., 2015) with ClinGen Sequence Variant Interpretation recommendations (Abou Tayoun et al., 2018). A total of 160 unique pathogenic or likely pathogenic *GATA2* variants were identified, with an additional 19 describing partial or complete *GATA2* gene deletions, resulting in haploinsufficiency. Phenotype expansion associated with G2DS has resulted in the duplication of individuals in the literature carrying germline *GATA2* variants, as more clinical and genetic information has become available. In this review, we have attempted to remove duplication by combining genetic and clinical data for these individuals as extrapolated from the referenced publications. A total of 480 individuals carrying a germline *GATA2* pathogenic or likely pathogenic variant were identified (Tables 1 and S1, Figures 1 and 2B). The available published data confirmed 240 individuals with inherited variants and 24 individuals carrying de novo variants. For the remaining 221 individuals with a G2DS phenotype, insufficient evidence was provided to conclude on the mode of *GATA2* variant acquisition. Classified variants of uncertain significance (VUS) or likely benign were excluded from analysis, but are provided in Table S2. *GATA2* pathogenic and likely pathogenic variants (ACMG/AMP classified) are very rarely seen in the general population (only 3 in total in gnomAD v2.1.1 and 3.0).

**Table 1:** GATA2 germline variants reported in families or individuals displaying one or more GATA2 deficiency syndrome (G2DS) phenotypes

Number individuals	Familial (F)/de novo (D)	GATA2 protein (NP_116027.2)	GATA2 cDNA (NM_032638.5)	Mutation effect	ACMG/AMP classification (curated)	ACMG/AMP criteria	Overall Phenotype	Chromosomal Abnormalities
1	N/A	p.(Glu6Alafs*178)	c.17_18del	FS	Pathogenic	PVS1, PS4_Supporting, PM2	*M/A*	−7
1	F	p.(Gln20*)	c.58C>T	NS	Pathogenic	PVS1, PS4_Supporting, PM2	*T‐ALL, HA, ID*	45,XX,dic(21;22)(p11.2;p11.2)
7	F	p.(Gly28Alafs*52) (in cis p.(His26Pro))	c.77A>C/c.83delG	FS	Pathogenic	PVS1, PS4, PM2, PP1_Moderate	*M/A, ID, L, HA*	−7
3	F	p.(Glu44*)	c.130G>T	NS	Pathogenic	PVS1, PS4_Moderate, PM2	*L, HA, ID*	
1	N/A	p.(Ser54*)	c.161C>A	NS	Pathogenic	PVS1, PS4_Supporting, PM2	*M/A*	−7
1	F	p.(Tyr59*) (in cis p.?)	c.177C>G (in cis c.[140T>G;142T>C;145T>C])	NS	Pathogenic	PVS1, PS4_Supporting, PM2	*M/A, ID*	+8
1	D	p.(Arg69Leufs*115)	c.206_208delGCGinsT	FS	Pathogenic	PVS1, PS2, PS4_Supporting, PM2	*M/A*	
1	N/A	p.(Val70Leufs*114)	c.207_208delCG	FS	Pathogenic	PVS1, PS4_Supporting, PM2	*M/A*	der(1;7) add
1	D	p.?	c.222_229+6del14ins21	INDEL	Pathogenic	PVS1, PS2, PS4_Supporting, PM2	*M/A*	+8
2	F	p.?	c.229+13_229+14insGCCins203_229+13	INDEL	Likely Pathogenic	PS4_Moderate, PM2, PP3	*M/A, ID*	+8
3	F	p.(Arg78Profs*107)	c.232dup [c.230‐1_230insC]	FS	Pathogenic	PVS1, PS4_ Moderate, PM2	*M/A, L, HA*	−7
1	N/A	p.(Gly82Argfs*103)[G81fs*]	c.243delinsGC	FS	Pathogenic	PVS1, PS4_Supporting, PM2	*M/A, ID, HA*	NK
1	N/A	p.(Arg86Profs*98) [C85fs*]	c.257_258del	FS	Pathogenic	PVS1, PS4_Supporting, PM2	*M/A, ID*	−7
1	N/A	p.(Arg86Alafs*33)	c.256del	FS	Pathogenic	PVS1, PS4_Supporting, PM2	*ID, M/A, HA*	
1	N/A	p.(Gly101Alafs*18) [G101Afs*16]	c.302del	FS	Pathogenic	PVS1, PS4_Supporting, PM2	*M/A, CMML, ID, HA*	del(11)(q13q23), −7, +8
3	N/A	p.(Ala103Glnfs*16)	c.303del	FS	Pathogenic	PVS1, PS4_ Moderate, PM2	*M/A*	−7, der(1;7)(q10;p10), der(1;7) add
1	N/A	p.(Ala103Glnfs*16)	c.306del	FS	Pathogenic	PVS1, PS4_Supporting, PM2	*M/A*	−7, add
5	F	p.(Leu105Profs*15)	c.312_313dup	FS	Pathogenic	PVS1, PS4, PM2	*M/A, ID, L, HA*	‐7
1	N/A	p.(Ser106Cysfs*78)	c.317_318del	FS	Pathogenic	PVS1, PS4_Supporting, PM2	*ID*	NK
2	F	p.(Ala107Cysfs*78) [S106fs]	c.318dup [c.318_319insT]	FS	Pathogenic	PVS1, PS4_ Moderate, PM2	*ID, L*	
15	F	p.(Thr117=)	c.351C>G	SPL	Pathogenic	PS3_Very Strong, PS4, PM2, PP1_Strong, PP3	*M/A, ID, L, HA, NS*	−7, +8, Chr1 translocation
1	N/A	p.(Val118Glyfs*100) [V618fs]	c.353del	FS	Pathogenic	PVS1, PS4_Supporting, PM2	*M/A*	−7
1	N/A	p.(Gly136Argfs*49)	c.404dup	FS	Pathogenic	PVS1, PS4_Supporting, PM2	*M/A, ID*	
1	N/A	p.(Ser139Cysfs*78)	c.414_417del	FS	Pathogenic	PVS1, PS4_Supporting, PM2	*ID, M/A, HA, L*	
1	N/A	p.(Ser139Cysfs*45)	c.416_417del	FS	Pathogenic	PVS1, PS4_Supporting, PM2	*M/A*	−7
1	N/A	p.(Val140Cysfs*45) [V140Cfs*44]	c.417dup	FS	Pathogenic	PVS1, PS4_Supporting, PM2	*M/A, ID, HA*	NK
2	F	p.(Tyr141*)	c.423C>A	NS	Pathogenic	PVS1, PS4_ Moderate, PM2	*M/A, ID*	−7
1	F	p.(Gly146Valfs*72)	c.437del	FS	Pathogenic	PVS1, PS4_Supporting, PM2	*M/A, ID*	NK
1	N/A	p.(Glu180*)	c.538G>T	NS	Pathogenic	PVS1, PS4_Supporting, PM2	*M/A, ID*	NK
1	N/A	p.(Thr188Hisfs*14)	c.561dup	FS	Pathogenic	PVS1, PS4_Supporting, PM2	*M/A*	NK
1	D	p.(Ala194Serfs*8)	c.579dup	FS	Pathogenic	PVS1, PS2, PS4_Supporting, PM2	*L, M/A*	−7
1	N/A	p.(Ala198Glyfs*20)	c.593del	FS	Pathogenic	PVS1, PS4_Supporting, PM2	*HA, ID*	
1	F	p.(Gly199Leufs*22)	c.586_593dup	FS	Pathogenic	PVS1, PS4_Supporting, PM2	*M/A, ID, HA*	−9q
5	F	p.(Gly200Valfs*18) [G199fs*]	c.599del [c.594delG]	FS	Pathogenic	PVS1, PS4, PM2	*M/A, ID, L*	−7
11	F/D	p.(Ser201*) [G200fs]	c.599dup [c.599_600insG]	NS	Pathogenic	PVS1, PS4, PM2	*M/A, ID, HA, L*	+8, −7
1	N/A	p.(Arg204*)	c.610C>T	NS	Pathogenic	PVS1, PS4_Supporting, PM2	*M/A, ID, HA*	46,XX,der(9)t(1;9)(q12;q12), r(9)(q12q?34)[15]/46,XX [9]
1	N/A	p.(Val211Argfs*72)	c.627_630dup	FS	Pathogenic	PVS1, PS4_Supporting, PM2	*M/A*	−7
1	N/A	p.(Glu224*)	c.670G>T	NS	Pathogenic	PVS1, PS4_Supporting, PM2	*M/A, ID, HA*	NK
1	N/A	p.(Leu229Cysfs*5)	c.685del	FS	Pathogenic	PVS1, PS4_Supporting, PM2	*M/A*	−7
1	N/A	p.(Gly237Alafs*89) [M236Ifs325*]	c.710del [c.708delC]	FS	Pathogenic	PVS1, PS4_Supporting, PM2	*HA, L, ID*	
2	N/A	p.(Ile246Hisfs*36) [P245fs*]	c.735dup [c.735_736insC]	FS	Pathogenic	PVS1, PS4_ Moderate, PM2	*ID, M/A, HA*	
1	F	p.(Tyr260Cysfs*25) [Y260fs*24, D259fs*]	c.769_778dup	FS	Pathogenic	PVS1, PS4_Supporting, PM2	*M/A, ID, HA*	−7
1	N/A	p.(Phe265Glufs*58)	c.793_802del	FS	Pathogenic	PVS1, PS4_Supporting, PM2	*M/A*	
3	F	p.(Gly268*)	c.802G>T	NS	Pathogenic	PVS1, PS4_ Moderate, PM2	*M/A, ID, L*	−13q ‐7 +8
2	N/A	p.(Gly273Thrfs*8)	c.817_818del [c.814_815del]	FS	Pathogenic	PVS1, PS4_ Moderate, PM2	*M/A*	‐7 +8
1	N/A	p.(Pro274Thrfs*8) [G273fs*]	c.818dupG [c.819insG]	FS	Pathogenic	PVS1, PS4_Supporting, PM2	*M/A*	Other
1	F	p.(Ala286Val)	c.857C>T	MS	Likely pathogenic	PS3, PS4_Supporting, PM2, PP3	*CMML*	
1	N/A	p.(Ser290*)	c.869C>A	NS	Pathogenic	PVS1, PS4_Supporting, PM2	*HA/ID*	
3	F	p.(Cys298Leufs*86)	c.892dup	FS	Pathogenic	PVS1, PS4_ Moderate, PM2	*ID, M/A*	Chr1‐7 translocation +8
2	F	p.(Trp306Alafs*77)	c.915_916del	FS	Pathogenic	PVS1, PS4_ Moderate, PM2	*M/A, ID, HA*	−7
1	N/A	p.(Trp306*)	c.917G>A	NS	Pathogenic	PVS1, PS4_Supporting, PM2	*M/A, HA, L, ID*	−7
1	N/A	p.(Thr311Argfs*71)	c.932_937delinsG	FS	Pathogenic	PVS1, PS4_Supporting, PM2	*M/A*	−7
1	F	p.(His313Tyr)	c.937C>T	MS	Likely Pathogenic	PS4_Supporting, PM1, PM2, PP3	*M/A, ID*	−5, −7, +8, add10, −12, −18, −21
1	F	p.(Tyr314Cysfs*66)	c.941_951del	FS	Pathogenic	PVS1, PS4_Supporting, PM2	*M/A, ID*	−7
1	N/A	p.(Leu315Pro)	c.944T>C	MS	Likely Pathogenic	PS4_Supporting, PM1, PM2, PP3	*ID*	
1	N/A	p.(Ala318Thrfs*12)	c.941_951dup	FS	Pathogenic	PVS1, PS4_Supporting, PM2	*M/A, ID, HA*	−6
1	F	p.(Ala318Thr)	c.952G>A	MS	Pathogenic	PS3, PS4_Supporting, PM1, PM2, PP3	*M/A, ID, HA*	+1,der(1:7)(q10:p10), +8
1	F	p.(Cys319Serfs*5)	c.956_962del	FS	Pathogenic	PVS1, PS4_Supporting, PM2	*ID, HA*	
1	N/A	p.(His323Glnfs*61)	c.968dup	FS	Pathogenic	PVS1, PS4_Supporting, PM2	*M/A*	−7
1	N/A	p.(Gln328*)	c.982C>T	MS	Pathogenic	PVS1, PS4_Supporting, PM2	*M/A, ID*	dic(1;15)(?;?)
13	F/D	p.(Arg330*)	c.988C>T	NS	Pathogenic	PVS1, PS4, PM2	*M/A, ID, L, HA, JMML*	−7, +8, +1
1	N/A	p.(Leu332Thrfs*53)	c.989_992dup [c.992_993insGACC]	FS	Pathogenic	PVS1, PS4_Supporting, PM2	*M/A, HA, L*	−7, +8
1	N/A	p.(Leu332Glnfs*60)	c.970_994dup	FS	Pathogenic	PVS1, PS4_Supporting, PM2	*M/A*	−7
7	F	p.(Arg337*)	c.1009C>T	NS	Pathogenic	PVS1, PS4, PM2	*M/A, ID, L, HA*	−7
1	N/A	p.?	c.1017+1del	SPL	Pathogenic	PVS1, PS4_Supporting, PM2	*M/A*	−7
2	N/A	p.? [S340Gfs*99]	c.1017+2T>G	SPL	Pathogenic	PVS1, PS4_ Moderate, PM2	*M/A, ID, HA*	−7
1	D	p.? [S340Afs*49]	c.1017+2T>C	SPL	Pathogenic	PVS1, PS2, PS4_Supporting, PM2	*M/A, HA, L, ID*	
2	F	p.? INT enhancer (del ETS site)	c.1017+513_1017+540del (c.1017+512del28)	REG	Likely Pathogenic	PS3_Supporting, PS4_ Moderate, PM1, PM2	*M/A, ID, HA*	
30	F	p.? INT enhancer (ETS site)	c.1017+572C>T	REG	Pathogenic	PS3, PS4, PM1, PM2, PP1_Strong	*M/A, ID, L, HA, CMML, ALL, B‐ALL*	−7 +8 +1 der(Y)t(Y;1)(q11.23;q21) der(1;7)(q10;p10)
1	N/A	p.?	c.1018‐11_1027del	SPL	Pathogenic	PVS1, PS4_Supporting, PM2	*M/A*	−7
2	N/A	p.?	c.1018‐10_1037del	SPL	Pathogenic	PVS1, PS4_ Moderate, PM2	*M/A*	−7
2	N/A	p.? [A341Rfs*38]	c.1018‐3_1031del	SPL	Pathogenic	PVS1, PS4_ Moderate, PM2	*ID, L, HA, HL*	
2	F	p.?	c.1018‐2A>G	SPL	Pathogenic	PVS1, PS4_ Moderate, PM2	*M/A, L*	−7
1	N/A	p.?	c.1018‐2A>T	SPL	Pathogenic	PVS1, PS4_Supporting, PM2	*M/A*	−7
2	N/A	p.? [S340‐N381del]	c.1018‐1G>A	DEL	Likely Pathogenic	PVS1_Strong, PS4_ Moderate, PM2	*M/A,HA, ID*	der(22)t(1;22)(q12;p13)/der(15)t(1;15)(q12;p13)
1	N/A	p.? [S340‐N381del]	c.1018‐1G>T	DEL	Likely Pathogenic	PVS1_Strong, PS4_Supporting, PM2	*HA,ID*	
2	F	p.(Ser340Lysfs*40)	c.1018_1028del	FS	Pathogenic	PVS1, PS4_ Moderate, PM2	*M/A, ID*	NK
1	N/A	p.(Ser340Trpfs*47)	c.1019del	FS	Pathogenic	PVS1, PS4_Supporting, PM2	*M/A*	−7
1	N/A	p.(Ala341Aspfs*53)	c.1019_1020insCGACTGGGAGGGCAAGGCAG	FS	Pathogenic	PVS1, PS4_Supporting, PM2	*M/A, HA, L, ID*	
1	N/A	p.(Ala341Profs*46)	c.1021del	FS	Pathogenic	PVS1, PS4_Supporting, PM2	*HA, ID, L*	
1	N/A	p.(Ala341Profs*45)	c.1021_1024del [c.1019_1022del]	FS	Pathogenic	PVS1, PS4_Supporting, PM2	*L, M/A, HL*	
3	F	p.(Ala341Serfs*39)	c.1021_1031del	FS	Pathogenic	PVS1, PS4_ Moderate, PM2	*M/A, ID*	−7, +8
1	N/A	p.(Ala342Argfs*42)	c.1023dup	FS	Pathogenic	PVS1, PS4_Supporting, PM2	*M/A, ID*	NK
2	F	p.(Ala342Profs*45)	c.1023del	FS	Pathogenic	PVS1, PS4_ Moderate, PM2	*ID, L*	NK
2	N/A	p.(Ala342del)l [341delA]	c.1024_1026del [c.1021_1023]	DEL	Pathogenic	PVS1, PS4_ Moderate, PM2	*M/A*	−7
1	N/A	p.(Arg343Profs*42) [A342Gfs*41]	c.1025_1026insGCCG	FS	Pathogenic	PVS1, PS4_Supporting, PM2	*ID, HA*	
2	F	p.(Arg344Glnfs*41)	c.1023_1026dup	FS	Pathogenic	PVS1, PS4_ Moderate, PM2	*M/A, ID, HA*	−7
3	N/A	p.(Arg344Glyfs*43)	c.1020_1029dup	FS	Pathogenic	PVS1, PS4_ Moderate, PM2	*M/A, ID, L, HA*	NK
1	D	p.(Arg344Lysfs*37)	c.1031_1049del	FS	Pathogenic	PVS1, PS2, PS4_Supporting, PM2	*M/A*	NK
1	N/A	p.(R344Kfs*40)	N/A	FS	Pathogenic	PVS1, PS4_Supporting, PM2	*M/A*	NK
1	N/A	p.(Gly346Serfs*40)	c.1035_1036ins TCTGGCC	FS	Pathogenic	PVS1, PS4_Supporting, PM2	*M/A, ID, HA*	
1	D	p.(Thr347Argfs*38)	c.1035_1038dup	FS	Pathogenic	PVS1, PS2, PS4_Supporting, PM2	*M/A*	−7
1	N/A	p.(Thr347Argfs*42)	c.1023_1038dup (c.1038_1039insCGCCAGAAGAGCCGGC)	FS	Pathogenic	PVS1, PS4_Supporting, PM2	*M/A*	−7
2	F	p.(T347fs)	16bp tandem dup	FS	Pathogenic	PVS1, PS4_ Moderate, PM2	*ID, HA*	
1	N/A	p.(Cys348Valfs*39)	c.1041del	FS	Pathogenic	PVS1, PS4_Supporting, PM2	*ID, M/A, HA*	−7
1	D	p.(Cys349Phe) [C348F]	c.1046G>T	MS	Likely Pathogenic	PS2, PS4_Supporting, PM1, PM2, PM5, PP3	*M/A*	−7
1	D	p.(Cys349Arg)	c.1045T>C	MS	Pathogenic	PS2, PS4_Supporting, PM1, PM2, PM5, PP3	*M/A, ID*	46,XY,der(3)t dic(1;3)(p11; p25)
1	N/A	p.(Cys349Gly)	c.1045T>G	MS	Likely Pathogenic	PS4_Supporting, PM1, PM2, PM5, PP3	*ID, M/A,L*	
1	N/A	p.(Asn351Ser)	c.1052A>G	MS	Likely Pathogenic	PS4_Supporting, PM1, PM2, PP3	*M/A*	−7
1	F	p.(Cys352Arg)	c.1054T>C	MS	Likely Pathogenic	PS4_Supporting, PM1, PM2, PM5, PP3	*M/A*	
1	N/A	p.(Cys352Gly)	c.1054T>G	MS	Likely Pathogenic	PS4_Supporting, PM1, PM2, PM5, PP33	*M/A*	−7
1	N/A	p.(Cys352Valfs*35)	c.1054del	FS	Pathogenic	PVS1, PS4_Supporting, PM2	*ID, M/A, HA, L*	NK
1	N/A	p.(Cys352Phe)	c.1055G>T	MS	Likely Pathogenic	PS4_Supporting, PM1, PM2, PM5, PP3	*M/A*	der(1;7)(q10;p10),+1
2	F/D	p.(Thr354Pro)	c.1060A>C	MS	Pathogenic	PS2, PS4_Moderate, PM1, PM2, PM5, PP3	*M/A, ID, L, HA*	NK
53	F	p.(Thr354Met)	c.1061C>T	MS	Pathogenic	PS3, PS4, PM1, PM2, PM5, PP1_Strong, PP3	*M/A, ID, L, HA*	−7, +8, +21, −5q, 1q abnormality, isochromosome 17, F100 t(1q:7p) +8 replaced by monodicentric 6
1	F	p.(Thr354Arg)	c.1061C>G	MS	Likely Pathogenic	PS4_Supporting, PM1, PM2, PM5, PP3	*M/A, ID*	46,XX [20]/92,XXXX [2]
2	F	p.(Thr358del) [355delT]	c.1065_1067del [c.1063_1065delACA]	DEL	Pathogenic	PS3, PS4_Moderate, PM2, PM4	*M/A*	−7 +8
1	N/A	p.(Thr358del)	c.1072_1074del	DEL	Pathogenic	PS3, PS4_Moderate, PM2, PM4	*ID*	
1	N/A	p.(Thr356_Asn365del)	c.1066_1095del	DEL	Likely Pathogenic	PS4_Supporting, PM2, PM4_Strong	*M/A*	−7
2	N/A	p.(Thr357Ala)	c.1069A>G	MS	Likely Pathogenic	PS4_ Moderate, PM1, PM2, PM5, PP3	*M/A*	der(1;7), add +8
1	N/A	p.(Thr357Ile)	c.1070C>T	MS	Likely Pathogenic	PS4_Supporting, PM1, PM2, PM5, PP3	*M/A, ID*	NK
1	F	p.(Thr358Asn), p.(Leu359Val) (in‐cis)	c.1073C>A c.1075T>G	MS	Pathogenic/VUS	PS3, PS4_Supporting, PM1, PM2, PP3/PM1, PM2, PM5, PP3, BP2	*M/A, HA, ID*	
1	F	p.(Leu359Val)	c.1076T>C	MS	Likely Pathogenic	PS4_Supporting, PM1, PM2, PP3	*M/A, ID, L*	46,XX,del(5)(q2?3q 3?3) [18]/46,XX
3	F	p.(Trp360Arg)	c.1078T>A	MS	Likely Pathogenic	PS4_Moderate, PM1, PM2, PM5, PP3	*ID, L, HA*	
1	F	p.(Trp360Leu)	c.1079G>T	MS	Likely Pathogenic	PS4_Supporting, PM1, PM2, PM5, PP3	*M/A, HA, ID*	−7
2	F	p.(Arg361Gly)	c.1081C>G	MS	Likely Pathogenic	PS4_Moderate, PM1, PM2, PM5, PP3	*M/A, ID, L*	
7	F	p.(Arg361Cys)	c.1081C>T	MS	Likely Pathogenic	PS4, PM1, PM5, PP3	*MA, ID, L, HA*	−7
1	D	p.(Arg361Leu)	c.1082G>T	MS	Pathogenic	PS2, PS3, PS4_Supporting, PM1, PM2, PM5, PP3	*L, HA, ID*	
8	F	p.(Arg361His)	c.1082 G>A	MS	Pathogenic	PS4, PM1, PM2, PM5, PP3	*M/A, NS, ID*	−7, +8
1	N/A	p.(Arg362_Asn365 del) [R361del4]	c.1084_1095del [c.1083_1094del12]	DEL	Likely Pathogenic	PS4_Supporting, PM2, PM4_Strong	*M/A, ID, HA*	−7, +21, +8
17	F/D	p.(Arg362*)	c.1084C>T	NS	Pathogenic	PVS1, PS4, PM2	*M/A, ID, L, HA*	−7, add +8 −20q
2	F	p.(Arg362Pro)	c.1085G>C	MS	Likely Pathogenic	PS4_ Moderate, PM1, PM2, PP3	*M/A, ID, L*	−7 +8
1	N/A	p.(Asp367Thrfs*20)	c.1099del	FS	Pathogenic	PVS1, PS4_Supporting, PM2	*M/A, HA, ID*	
3	F	p.(Asp367Glyfs*17)	c.1099dup [c.1099insG]	FS	Pathogenic	PVS1, PS4_ Moderate, PM2	*M/A, ID, HA*	NK
1	N/A	p.(Pro368Argfs*15)	c.1103_1104del	FS	Pathogenic	PVS1, PS4_Supporting, PM2	*NS*	
1	N/A	p.(Cys370Trp)	c.1110C>G	MS	Likely Pathogenic	PS4_Supporting, PM1, PM2, PP3	*M/A*	−7
4	N/A	p.(Asn371Lys)	c.1113C>A	MS	Pathogenic	PS1, PS4, PM1, PM2, PP3	*MA, L, ID*	−7 +8
2	N/A	p.(Asn371Lys)	c.1113C>G	MS	Pathogenic	PS1, PS4_Moderate, PM1, PM2, PP3	*M/A, HA,ID*	−7, +mar
1	N/A	p.(Asn371Lysfs*16)	c.1113del	FS	Pathogenic	PVS1, PS4_Supporting, PM2	*M/A*	−7, +8
6	F	p.(Ala372Thr)	c.1114G>A	MS	Likely Pathogenic	PS4, PM1, PM2, PP3	*M/A, ID, L*	−7, +15, +20 t(11;19), +8
3	F	p.(Cys373_Tyr377del) [C373del5]	c.1117_1131del [c.1116_1130del15]	DEL	Likely Pathogenic	PS4_ Moderate, PM2, PM4_Strong*	*M/A, ID*	−7
1	N/A	p.(Cys373Tyr)	c.1118G>A	MS	Likely Pathogenic	PS4_Supporting, PM1, PM2, PM5, PP3	*M/A, ID, L*	+1 −15
1	N/A	p.(Cys373Arg)	c.1117T>C	MS	Likely Pathogenic	PS3_Supporting, PS4_Supporting, PM1, PM2, PM5, PP3	*L, M/A, ID*	−7
1	N/A	p.(Leu375Val), p.?	c.1123C>G in‐cis with 355 bp of GATA2 locus (part of last intron & exon) that has been duplicated and inserted into the last exon	MS, INS	Likely Pathogenic	PM1, PM2, PM5, PP3	*M/A*	NK
3	F	p.(Leu375Phe)	c.1123C>T	MS	Likely Pathogenic	PS4_Moderate, PM1, PM2, PM5, PP3	*M/A, ID, L, HA*	−7, +8, +20
2	D	p.(Leu375Profs*12)	c.1124del	FS	Pathogenic	PVS1, PS2, PS4_ Moderate, PM2	*M/A*	−7
1	D	p.(Tyr376*)	c.1128C>G	NS	Pathogenic	PVS1, PS2, PS4_Supporting, PM2	*M/A*	NK
1	N/A	p.(Tyr377Asp)	c.1129T>G	MS	Likely Pathogenic	PS4_Supporting, PM1, PM2, PP3	*ID, HA*	
1	N/A	p.(Lys378*)	c.1132A>T	NS	Pathogenic	PVS1, PS4_Supporting, PM2	*HA, ID*	hyperdiploidy (>80 chr), +8
2	F	p.(Lys378Asnfs*12)	c.1126_1133dup	FS	Pathogenic	PVS1, PS4_ Moderate, PM2	*HA, ID, M/A*	NK
1	N/A	p.(Asn381Metfs*6)	c.1142del	FS	Pathogenic	PVS1, PS4_Supporting, PM2	*M/A, ID*	NK
1	D	p.? [S340_N381del, V382Gfs*23 or N381_V382ins41]	c.1143+2T>A	SPL	Pathogenic	PVS1, PS2, PS4_Supporting, PM2	*M/A, ID, HA*	+8
3	D	p.? [N381fs*20]	c.1143+5G>A	SPL	Likely Pathogenic	PS3, PS4_Moderate, PM2, PM6_Supporting	*ID, M/A*	
2	N/A	p.?	c.1143+5G>C	SPL	Likely Pathogenic	PS1_Supporting, PS4_Moderate, PM2, PP3	*M/A, ID*	+8
1	N/A	p.? [N381fs*]	c.1143+200_1198del	DEL	Pathogenic	PVS1, PS4_Supporting, PM2	*HA, ID, L*	NK
3	F	p.(Pro385Gln)	c.1154C>A	MS	Likely Pathogenic	PS4_ Moderate, PM1, PM2, PP3	*M/A, ID, L, T‐ALL*	−7
1	N/A	p.(Thr387Asn)	c.1160C>A	MS	Likely Pathogenic	PS4_Supporting, PM1, PM2, PP3	*M/A*	+8
2	F	p.(Met388Val)	c.1162A>G	MS	Likely Pathogenic	PS4_ Moderate, PM1, PM2, PM5, PP3	*M/A, HA, ID*	−7 +8
5	F	p.(Met388Thr)	c.1163T>C	MS	Likely Pathogenic	PS4, PM1, PM2, PM5, PP3	*ID, HA*	
1	N/A	p.(Lys390Glu)	c.1168A>G	MS	Likely Pathogenic	PS4_Moderate, PM1, PM2, PP3	*M/A*	der(1;7) add
1	N/A	p.(Glu391Glyfs*85)	c.1172_1175del	FS	Pathogenic	PVS1, PS4_Supporting, PM2	*ID, HA*	
12	F	p.(Arg396Trp)	c.1186C>T	MS	Pathogenic	PS4, PM1, PM2, PM5, PP3	*M/A, ID, L, HA*	+8, +mar, −7, −21
24	F/D	p.(Arg396Gln)	c.1187G>A	MS	Pathogenic	PS2, PS3, PS4, PM1, PM2, PM5, PP1_Moderate, PP3	*M/A, ID, L, HA*	+8, −7, +11, der(1;16), add
3	F	p.(Arg396Leu)	c.1187G>T	MS	Likely Pathogenic	PS3_Supporting, PS4_ Moderate, PM1, PM2, PM5, PP1_Supporting, PP3	*M/A, HA, ID*	
22	F	p.(Arg398Trp)	c.1192C>T	MS	Pathogenic	PS3, PS4, PM1, PM2, PM5, PP1_Moderate, PP3	*M/A, ID, HA, CMML, JMML, L*	+1, −7, +8, ‐X
4	F	p.(Arg398Gln)	c.1193G>A	MS	Likely Pathogenic	PS4, PM1, PM5, PP3	*M/A, ID*	−7, +8
1	N/A	p.(Lys406Serfs*77)	c.1200_1216dup [c.1216_1217ins17]	FS	Pathogenic	PVS1, PS4_Supporting, PM2	*M/A*	−7
1	N/A	p.(Phe428Leufs*108)	c.1281dup [c.1281_1282insC]	FS	Pathogenic	PVS1, PS4_Supporting, PM2	*M/A*	
1	N/A	p.(His442Glnfs*95)	c.1322_1325dup	FS	Likely pathogenic	PVS1_Moderate, PS4_Supporting, PM2	*M/A*	
8	F	p.(Ser447Arg)	c.1339A>C	MS	Pathogenic	PS1, PS4, PM2, PP1_Moderate, PP3	*M/A, ID, HA*	+8
2	N/A	p.(Ser447Arg)	c.1341C>A	MS	Likely pathogenic	PS1, PS4_Moderate, PM2	*M/A*	−7
1	N/A	Deletion (whole protein)		DEL	Pathogenic	PVS1, PS4_Supporting, PM2	*L, M/A, ID, DF, NS*	+21
1	N/A	Deletion (whole protein)		DEL	Pathogenic	PVS1, PS4_Supporting, PM2	*M/A, ID, DF, NS, HA*	
1	N/A	Deletion (whole protein)		DEL	Pathogenic	PVS1, PS4_Supporting, PM2	*HA, ID*	
1	N/A	Deletion (whole protein)		DEL	Pathogenic	PVS1, PS4_Supporting, PM2	*ID, HA*	
2	N/A	Deletion (whole protein)	c.1‐?_1443+?del	DEL	Pathogenic	PVS1, PS4_ Moderate, PM2	*M/A, ID*	NK
1	N/A	Deletion (whole protein)		DEL	Pathogenic	PVS1, PS4_Supporting, PM2	*M/A, ID, DF, NS, HA*	−7
1	N/A	Deletion (whole protein)		DEL	Pathogenic	PVS1, PS4_Supporting, PM2	*M/A, HA, ID, L*	−7, +8
1	N/A	Deletion (whole protein)		DEL	Pathogenic	PVS1, PS4_Supporting, PM2	*M/A, HA, ID*	
1	N/A	Deletion (whole protein)		DEL	Pathogenic	PVS1, PS4_Supporting, PM2	*M/A*	−7
1	N/A	del3q21		DEL	Pathogenic	PVS1, PS4_Supporting, PM2	*M/A, ID, NS*	del3q21, −7
1	N/A	Deletion		DEL	Pathogenic	PVS1, PS4_Supporting, PM2	*M/A, HA, ID*	45,XY,‐7[7]/46,XY,‐7,+mar[10]/46,XY[8]
1	N/A	Deletion		DEL	Pathogenic	PVS1, PS4_Supporting, PM2	*HA, ID*	
1	F	Delete ATG start codon		DEL	Pathogenic	PVS1, PS4_Supporting, PM2	*M/A*	t(2;12)(p21;p13)
3	F	p.? (M1del290)	c.‐45‐155_871+527del	DEL	Pathogenic	PVS1, PS4_ Moderate, PM2	*M/A, ID, L, HA*	NK
1	N/A	p.?		DEL	Pathogenic	PVS1, PS4_Supporting, PM2	*M/A, HA*	
1	F	p.?		DEL	Pathogenic	PVS1, PS4_Supporting, PM2	*M/A*	−7
1	F	p.?		DEL	Pathogenic	PVS1, PS4_Supporting, PM2	*M/A, ID*	(7q22)x1,(7q31)x1 [260/400]
1	F	del ZF2 & C‐terminus	c.1018‐?	DEL	Pathogenic	PVS1, PS4_Supporting, PM2	*M/A, ID*	
1	N/A	del ZF2 & C‐terminus	c.1018‐?_1443+?del	DEL	Pathogenic	PVS1, PS4_Supporting, PM2	*ID, M/A*	

*Note*: This table summarizes germline GATA2 likely pathogenic/pathogenic variants with full details available in Table S1. All mutations are numbered from the ATG start codon of GATA2 NM_032638.5 and NP_116027.2. Where the original published nomenclature differs, the original published mutation is indicated in square brackets []. For each mutation, the observed phenotypes for each individual were combined to generate the overall phenotype. Overall phenotypes: CMML, chronic myelomonocytic leukemia; DF, dysmorphic features; HA, hematological abnormality; HL, hearing loss; ID, immunodeficiency; JMML, juvenile myelomonocytic leukemia; L, lymphedema; M/A, MDS and/or AML; NS, neurologic symptoms; T‐ALL, T‐cell acute lymphoblastic leukemia. Mutation effect: DEL, large deletion; FS, frameshift; INDEL, small insertion/deletion; MS, missense; NS, nonsense; REG, regulatory; SPL, splice site.

### 1.2 *GATA2* cohort characteristics

In the literature, 359 different families with germline *GATA2* variants have been reported which encompasses 179 different variants (Tables 1 and Table S1, Figures 1 and 2A). There is an equal representation of both males and females presenting with G2DS phenotypes (1:1.08). In the MDS and MDS/AML population, the gender ratio was 1:1.04 suggesting no gender bias is associated with this mechanism of predisposition. This is interesting given a male bias of 1.8:1 for sporadic adult MDS (Greenberg et al., 2012; Stauder et al., 2018) and ~6:1 for germline DDX41‐driven MDS (Lewinsohn et al., 2016; Polprasert et al., 2015; Quesada et al., 2019; Sébert et al., 2019), and likely reflects the earlier onset of G2DS MDS associated with mechanistic specificities of disease pathobiology between different drivers of malignancy. The median age of GATA2‐driven myeloid malignancy (GDMM) onset is 17 years, with onset ranging from 0 to 78 years of age, with ~75% of identified *GATA2* carriers developing a malignancy. The median age of onset is marginally earlier in males as compared to females (17 and 19, respectively). The majority of individuals presented with MDS (56.4%) followed by MDS/AML (9.12%) and AML (6.9%), with the median age of onset being 17 years for both MDS and MDS/AML and 20 years for AML. Four carriers presented with lymphoid malignancy (T‐ or B‐cell acute lymphoblastic leukemia), although this was rare in comparison to the myeloid malignancy (1.1% vs. 98.8%), and may represent sporadic cases. Notably, within this small lymphoid malignancy cohort, two individuals (50%) developed ALL with monosomy 7 (common in GDMM).

GATA2‐related immunodeficiency is also known as immunodeficiency 21 (MIM# 614172). These immunodeficient patients suffer from recurrent viral (Human *papillomavirus* leading to warts, Epstein‐Barr virus), fungal (commonly within the respiratory tract), and bacterial (most commonly Mycobacterial strains) infections, often as a result of various cytopenias, predominantly reduced or absent monocytes, B cells, NK cells, neutrophils, and/or dendritic cells (Spinner et al., 2014; Tangye et al., 2020). These patients are seen in primary immunodeficiency cohorts and are often treated with hematopoietic stem cell transplantation (HSCT) due to the severity of immunodeficiency. It is not clear if and to what extent such BM failure contributes to the development of GDMM (Arts et al., 2019; Spinner et al., 2014; Stray‐Pedersen et al., 2016).

The range in age of onset and penetrance of disease types, even within the same family, remains an intriguing mystery, one that if better understood may lead to beneficial prevention strategies. One possible answer is that of acquired monoallelic expression due to epigenetic silencing as a mechanism for further lowering of GATA2 activity levels (Al Seraihi et al., 2018).

### 1.3 Type of *GATA2* variants

The majority of unique germline pathogenic and likely pathogenic variants in *GATA2* seen in G2DS are loss‐of‐function (LOF) either through frameshift (40.2%), nonsense (10.1%), splicing (6.7%), or deletions (15.6%) throughout the gene. Missense variants (25.7%), mostly in the zinc finger 2 (ZF2) domain, also contribute a large proportion of the germline *GATA2* variants, likely disrupting DNA binding (Chong et al., 2018; Hahn et al., 2011) or protein–protein interactions (Bresnick et al., 2020; Chong et al., 2018). The other important group, accounting for 1.1% G2DS variants, are regulatory, which affect the *GATA2* intron 4 enhancer site and lead to reduced GATA2 expression in a range of hematopoietic stem and progenitor cells (Gao et al., 2013; Johnson et al., 2012, 2020; Mehta et al., 2017). Elegant studies in mice have shown the temporal and cell‐specific roles of the −77 kb and +9.5 kb (i.e., intron 4) *Gata2* enhancers in development and cell differentiation fates (Bresnick et al., 2020). Although the repositioning of the equivalent −77 kb *GATA2* enhancer in humans has been shown to be important in t(3;3)(q21;q26) and inv(3)(q21;q26) in AML in activating *MECOM* (Bresnick & Johnson, 2019; Gröschel et al., 2014; Yamaoka et al., 2020), no germline variants have been reported in this enhancer to date, possibly a result of this region not being included in most screening protocols.

Interestingly, there are only a few reports of confirmed missense germline variants in zinc finger 1 (ZF1). One of these is p.Ala318Thr (Kurata et al., 2017) which is seen 13 times as a somatic mutation in COSMIC (v 92) (also p.Ala318Asp/Gly/Val, 25 times) and occurs in a somatic mutational hotspot (p.Asn317‐Leu321, 95 times) with all in myeloid malignancies. In vitro transactivation studies demonstrated partial LOF (Greif et al., 2012; Katsumura et al., 2018). The p.Ala318Thr case was a 10‐year‐old girl with MDS and she had an unaffected mother and sister who were both carriers. Of note, a der(1;7)(q10;p10) was seen in this MDS case, which is a rare translocation that has been reported multiple times in pediatric/childhood (An et al., 2019; Wang et al., 2015; Wlodarski et al., 2016) and young adult MDS (Ganapathi et al., 2015) with pathogenic germline *GATA2* variants; this results in chromosome 7q deletion analogous to −7/7q that is the most common acquired cytogenetic event in GDMM (Brown et al., 2020). Other ZF1 variants include p.His313Tyr and p.Leu315Pro, where p.His313Tyr has typical G2DS phenotypes (Donadieu et al., 2018). Intriguingly, a recent study confirmed a germline p.Asn317Ser variant (somatic in COSMIC –4 times; p.Asn317Ile/His, 5 times) in the first reported case of GATA2‐related primary myelofibrosis, which later progressed to pancytopenia (Rütsche et al., 2021). It is not clear whether this germline *GATA2* variant contributed to this JAK2 positive myeloproliferative neoplasm and/or if it drove the pancytopenia. Notably, for each of these four ZF1 variants, there is only one affected individual. Hence, there is a small but growing number of germline GATA2 ZF1 variants associated with G2DS.

The only missense variant outside of ZF1 and the extended ZF2 domain is A286V that is predicted to impact splicing. p.Ala286Val has been shown to generate a cryptic splice donor site leading to a 16 nt deletion in RNA studies (Guidugli et al., 2017); hence this variant may better be described as a frameshift variant. In addition, a seemingly innocuous synonymous variant (p.Thr117=) has been shown by at least three groups to strongly alter splicing, generating a predominant transcript (Kozyra et al., 2020; Wehr et al., 2018) (and our unpublished data) that would result in the variant being more accurately called p.Val118Glnfs*55 (Fox et al., 2020). Hence, for some variants, there is an obvious disconnect between their annotation at the DNA level and their functional effect or impact. With the incorporation of appropriate bioinformatic tools (e.g., SpliceAI) into variant annotation pipelines, such cases can be highlighted, but the actual impact on splicing often requires functional studies for accurate interpretation.

Recurrent variants in the extended ZF2 domain have been reported including p.Thr354Met (53 individuals and 22 families), p.Arg396Trp (11 individuals and 11 families), p.Arg396Gln (24 individuals and 16 families), and p.Arg398Trp (22 individuals and 17 families). These variants seem predominantly to be passed on through familial inheritance although there are two cases of de novo p.Arg396Gln. Using haplotype mapping, p.Thr354Met was shown to be both a founder variant (with a common haplotype in an Australian and USA family) as well as independently derived in another family in the United States (Hahn et al., 2011). Notably, many of the most commonly seen germline variants represent potential mutational “hotspots.” For instance, recurrent *GATA2* variants (p.Thr354Met, p.Arg396Gln/Trp, p.Arg398Gln/Trp, p.Arg362*, p.Arg361His/Cys, c.1017+572C>T) represent C>T (or G>A) changes at CpG dinucleotides that may be generated by spontaneous or enzymatic deamination of 5‐methylcytosines deposited during normal gene silencing and regulation. Such mutations (Signature SBS1) are considered part of the “normal” aging process (Alexandrov et al., 2020), and may contribute to generation of de novo and “founder” mutations/variants.

This abundance of Signature SBS1‐like variants in germline *GATA2* differs from another myeloid malignancy predisposition gene, *DDX41*, where causal variants predominate within ethnicities or regions (e.g., p.Met1Ile/p.Met1? and p.Asp140Glyfs*2 in Europeans; p.Val152Gly, p.Ala500Cysfs*9 and p.Tyr259Cys in East Asians) (Choi et al., 2019; Kim et al., 2020; Qu et al., 2020; Yasuda et al., 2020) suggesting that these are derived from founder variants that are not actively selected against in the general population. This is consistent with the late age of onset of myeloid malignancy for *DDX41* predisposition. The often early age of onset of malignancy, immunodeficiency, or other phenotypes for G2DS and apparent anticipation seen in some *GATA2* families (Hahn et al., 2011) may explain a lower level of founder effect and lower predominance of germline *GATA2* variants in sporadic adult MDS or AML cohorts compared to *DDX41* (Kim et al., 2020; Qu et al., 2020; Quesada et al., 2019; Yasuda et al., 2020).

### 1.4 Genotype/phenotype correlations of germline *GATA2* pathogenic and likely pathogenic variants

Among the reported phenotypes associated with germline *GATA2* variants, myeloid malignancy is the most common phenotype (74.3%) with a median age of onset of 17 years (Figure 2c). This is in stark contrast with the median age of sporadic disease (MDS, 76 years; AML, 68 years (Appelbaum et al., 2006; Ma et al., 2007). Immunodeficiency was reported in 62% of affected carriers. All variant types are able to predispose to the three major phenotypes (Figure 2c).

To date, different types of *GATA2* variants have not been linked strongly to particular subtypes of myeloid malignancy or age of onset or clinical outcomes. Interestingly, there is a predominance of myeloid malignancy with p.Thr354Met (83%; 44/53 cases) and immunodeficiency with p.Arg398Trp/Gln variants (85% cases). It remains to be established if variable clinical presentation is intrinsic to particular variants or is a consequence of ascertainment bias from recruitment criteria of various patient cohorts or whether there may be more prolific local environmental stressors in communities in which some founder variants are more prevalent.

There have been observations for the requirement of haploinsufficiency for lymphedema development (Kazenwadel et al., 2012). The majority of variants reported in lymphedema patients are premature termination variants (mainly frameshift or nonsense) resulting in haploinsufficiency (Figure 2c). Missense variants associated with lymphedema are predicted to be LOF and have high REVEL scores (range 0.879–0.989, median REVEL score 0.954). In vitro DNA binding and transactivation assays on three of the missense variants found in Emberger syndrome (p.Arg361Leu, p.Cys373Arg, p.Arg396Gln) demonstrated complete or almost complete LOF due to markedly diminished DNA binding and transactivation capacity (Chong et al., 2018; Kazenwadel et al., 2015). In keeping with haploinsufficiency, of 52 individuals carrying p.Thr354Met (retains residual GATA2 activity) (Chong et al., 2018; Hahn et al., 2011), there are no reported cases of lymphedema apart from an isolated case of vulvar lymphedema (Álvarez‐Chinchilla et al., 2017). The reason that haploinsufficient GATA2 mutations cause lymphedema is due to a crucial role for GATA2 in the development and maintenance of lymphatic vessel valves (Kazenwadel et al., 2015). In the setting of valve dysfunction, lymph is not efficiently returned to the bloodstream and lymphedema ensues. Mechanical stimuli including fluid flow and extracellular matrix‐induced tension, to which endothelial cells are exposed, have been demonstrated to regulate GATA2 levels in lymphatic endothelial cells (Frye et al., 2018, Kazenwadel et al., 2015). Moreover, these distinct mechanical signals induce specific transcriptional outputs; while oscillatory flow drives lymphatic vessel valve morphogenesis (Kazenwadel et al., 2018), exposure of cells to soft matrix drives lymphatic vessel sprouting and migration (Frye et al., 2018). Although hematopoietic transcription factors including SCL/TAL1 (Janardhan et al., 2017), ERG (Kazenwadel and Harvey, unpublished), and LMO2 (Coma et al., 2013) are present in lymphatic endothelial cells, the specific effects of the *GATA2* mutations associated with Emberger syndrome on lymphatic vascular development suggest that unique transcriptional complexes comprising GATA2 are present in lymphatic endothelial cells compared to hematopoietic cells and that these are differentially impacted by distinct *GATA2* mutations. The identification of GATA2 interacting transcriptional components, together with the signals that regulate their assembly in distinct cell types, will provide novel insight to the mechanisms by which *GATA2* variants result in the varied phenotypes described in patients with G2DS.

Interestingly, certain *GATA2* variants have been shown to display LOF and gain‐of‐function in different contexts. Though p.Thr354Met and p.Cys373Arg both disrupt the ZF2 structure in a way that reduces or abolishes DNA binding, respectively, they display an increased affinity for a known GATA2‐binding partner protein SPI1 (PU.1) (Chong et al., 2018). This has implications for downstream targets of both proteins in normal hematopoiesis and may contribute to leukemogenesis via mechanisms in addition to simple LOF mutations. A similar situation has been reported for certain somatic *GATA2* mutations; p.Arg307Trp displays increased ability to skew towards granulocytic differentiation and induce cell cycle progression (Katsumura et al., 2018), and p.Leu359Val shows context‐dependent increased transactivation and DNA binding and the subsequent impact on downstream target genes (Chong et al., 2018; Hahn et al., 2011; Zhang et al., 2008).

In vitro differentiation studies of certain *GATA2* variants showed increased granulocytic differentiation and a concurrent reduction in macrophage differentiation consistent with monocytopenia (Chong et al., 2018; Cortés‐Lavaud et al., 2015) and a case of noninfectious granulomatous dermatosis in patients (Polat et al., 2018). Also, patients with GATA2*‐*driven MDS display better maintenance of neutrophil counts than unselected MDS patients (Collin et al., 2015) and GATA2 deficient patients display a skewing of B‐cell to T‐cell differentiation (Nováková et al., 2016).

### 1.5 Somatic mutations associated with germline GDMM

There are a number of recurrent somatic cytogenetic aberrations and gene mutations that are seen with germline GDMM. Cytogenetically, the most common is monosomy 7, deletion of 7q and to a lesser extent der(1;7)(q10;p10), all of which effectively results in a loss of one copy of 7q (at least 49.5% (141/285) cases collectively; note, not all cases reported cytogenetics). Interestingly, in childhood MDS, these cytogenetic events occur more frequently than in sporadic cases with monosomy 7 seen in 37% pediatric and 72% adolescent MDS with germline *GATA2* variants (Wlodarski et al., 2016) and der(1;7) is also more prevalent (Kurata et al., 2017; Wlodarski et al., 2016). Trisomy 8 is the next most common cytogenetic event occurring in at least 22.8% (65/285) of cases. Given the relative rarity of germline *GATA2* cases and the lack of routine screening for somatic mutations, estimation of the prevalence and range of somatic gene mutations and those that are mutually exclusive is likely to be only indicative. At the gene level, *ASXL1*, *NRAS*/*KRAS*, *STAG2*, and *SETBP1* are most commonly seen somatically mutated in MDS and AML. Interestingly, *SF3B1*, *U2AF1*, *NPM1*, and *FLT3* mutations are uncommon in GATA2‐driven AML. *FLT3* mutations may be rare as the FLT3 ligand is often elevated in symptomatic G2DS providing elevated stimulus for FLT3 signaling that may negate selection of spontaneous *FLT3* mutations (Dickinson et al., 2011, 2014).

Both monoallelic and biallelic *CEBPA* somatic mutations are often associated with somatic *GATA2* mutation in sporadic AML (Fasan et al., 2013; Greif et al., 2012). Although somatic *CEBPA* mutations have been reported in germline GDMM (5 cases, Table 1), because of the past difficulties in sequencing this highly GC‐rich gene, it is unclear as to the exact frequency of concurrent *CEBPA* mutations.

Somatic *GATA2* mutations rarely occur in GDMM (i.e., biallelic) unlike for germline *RUNX1*, *CEBPA*, and *DDX41*‐driven myeloid malignancies where biallelic mutations are common (Brown et al., 2020; Cheah et al., 2017). Notably, while germline *GATA2* premature termination mutations (frameshift, nonsense, and splice) and ZF2 variants are most common in GDMM, *GATA2* mutations in sporadic myeloid malignancies are predominantly missense (mainly in ZF1, but also throughout the C‐terminus) or in‐frame indels in the C‐terminus, and premature termination mutations are seen to a lesser extent (COSMIC v92). This suggests that there may be a fundamental difference in the role of mutant GATA2 in the leukemogenic process between germline cases where mutant GATA2 is present in all cells throughout development including in the BM microenvironment, and somatic cases where the mutant protein is confined to hematopoietic cells and is rarely the first mutation acquired (Martignoles et al., 2018).

There is a need for a more systematic and comprehensive screening of somatic mutations in these rare cases of GDMM to better understand the range and frequency of concurrently mutated genes and why mutations in mutually exclusive genes are not required or selected for.

### 1.6 Clinical and diagnostic relevance

Due to the often early onset of G2DS symptoms and the potential severity of these, it is important to screen for and identify *GATA2* germline pathogenic variants to help guide clinical decisions and facilitate family counseling. Although a family history of myeloid malignancies or immunodeficiency may help in the decision to screen for *GATA2* variants, it is important to note that GATA2 de novo variants constitute a significant proportion of cases in some phenotypes such as in children or young adults with MDS (Wlodarski et al., 2016). Interestingly, there is even a report of a de novo c.1143+5G>A variant occurring in monozygotic twins both with typical G2DS phenotypes (Stray‐Pedersen et al., 2016). Depending on the severity of the disease at the time of molecular diagnosis, there may be an urgent need for therapeutic intervention. There are numerous reports of the requirement for early HSCT for G2DS patients with immunodeficiency or myeloid malignancies (Cuellar‐Rodriguez et al., 2011; McReynolds et al., 2018).

Considering the variable phenotypes in G2DS, family cascade testing of an inherited pathogenic variant may identify carriers that are asymptomatic or have a subclinical presentation within the spectrum of the disorder. Since the first report of *GATA2* as a predisposition gene for myeloid malignancies in 2010 (Scott et al., 2010), screening for germline variants in relatives has been increasingly implemented to select for HSCT donors that do not carry the variant to avoid donor‐derived MDS/AML (Galera et al., 2018) or for individuals that may need surveillance for malignancy or immunodeficiency, and this is becoming routine in many clinics (DiNardo et al., 2016; University of Chicago Hematopoietic Malignancies Cancer Risk Team, 2016). In cases of myeloid malignancy, it is becoming widely recognized that different somatic mutations can have different prognostic values. In children and adolescents with MDS and monosomy 7, germline *GATA2* variants do not confer poorer outcomes (Wlodarski et al., 2016).

Currently, HSCT is the most common treatment for the blood‐related phenotypes in G2DS (immunodeficiency and myeloid malignancy). Considering the high risk of developing these conditions, providing early genetic diagnosis to patients and/or their relatives before onset could allow more time to identify optimal HSCT donors, and lead to improved outcomes of transplantations.

Although *GATA2* variants can predispose to G2DS symptoms, other influences may be important in expression of these variants such as epigenetics. One report provided evidence of monoallelic expression of the p.Thr354Met allele in symptomatic patients while asymptomatic individuals display equal expression from both p.Thr354Met and wildtype alleles (Al Seraihi et al., 2018). Such silencing of the wildtype allele may be selected to further reduce GATA2 activity to even more “favorably” low levels for aberrant cell expansion or survival under certain conditions. Whether the stimulus for such epigenetic changes is physiological or environmentally driven is unknown.

The ability to correct *GATA2* pathogenic variants using gene therapeutic or editing approaches, particularly in the hematopoietic system, is an attractive idea. A recent paper reported evidence of a spontaneous *GATA2* somatic genetic rescue event in the hematopoietic system of an elderly asymptomatic individual, opening up the exciting possibility of therapeutic strategies to facilitate and expedite correction of pathogenic variants to enable hematopoietic recovery or normalization (Catto et al., 2020).

### 1.7 In vivo models to dissect the functional role of GATA2 in hematopoietic and nonhematopoietic tissues

Before the discovery of GATA2 as a disease gene in humans, the first Gata2 knockout (KO*‐Gata2* ^−/−^) mice generated showed embryonic lethality at 10.5 days post coitum due to lack of definitive hematopoiesis and severe anemia (Tsai et al., 1994). Further characterization on mouse embryonic stem cells showed that GATA2 is required for proliferation of early hematopoietic stem cells (HSC) and that loss of GATA2 expression interrupts normal embryonic development (Tsai & Orkin, 1997; Tsai et al., 1994). Heterozygous *Gata2* ^+/−^ mice survive until adulthood and are normal and fertile despite reduced expression (~50%) of Gata2 compared to wild‐type mice (Tsai & Orkin, 1997; Tsai et al., 1994). In the setting of stress hematopoiesis, BM from *Gata2* ^+/^^−^ mice exhibited a reduction in the abundance and functionality of immunophenotypically defined HSC (Guo et al., 2013; Rodrigues et al., 2005) displaying a phenotype resembling patients with G2DS immunodeficiency (Brown et al., 2020). *Gata2* haploinsufficiency also reduced granulocyte‐macrophage progenitor (GMP) cell function while leaving other myeloid committed progenitors intact (Rodrigues et al., 2008). Together, these observations show that GATA2 plays a stage‐specific differentiation role not only in the HSC but also the GMP compartment and that germline *GATA2* LOF variants in humans may act similarly to predispose to distinct diseases such as MDS/AML, immunodeficiency, and lymphedema (Brown et al., 2020; Pasquet et al., 2013).

Interestingly, a study on the effect of hypomorphic *Gata2* variants in mice showed that reduction of GATA2 expression to ~20% induced development of chronic myelomonocytic leukemia‐like leukemia (Harada et al., 2019). Furthermore, animal models have shown the importance of the mouse *Gata2* −77 kb upstream and +9.5 kb intronic enhancers in hematopoietic cell production and differentiation in embryonic, fetal, and adult hematopoiesis (reviewed in Bresnick et al., 2020; Johnson et al., 2020; Soukup & Bresnick, 2020). Germline variants in the +9.5 kb enhancer in humans predispose to myeloid malignancies, aplastic anemia, immunodeficiency, and lymphedema (Hsu et al., 2013; Johnson et al., 2012).

Tissue‐specific conditional KO models of Gata2 have been used to interrogate its roles in various tissues during later stages of development. For instance, conditional KO of *Gata2* under control of VECre (i.e., VE‐Cadherin‐Cre: *Gata2* deletion in vascular endothelial cadherin‐expressing endothelial cells before HSC formation) and Vav‐Cre (*Gata2* deletion in hematopoietic cells after HSC generation) showed that complete deletion of *Gata2* in these tissues was embryonic lethal due to defects in endothelial to hematopoietic transition during HSC formation and HSC survival (de Pater et al., 2013). The VECre mediated Gata2 loss resulted in death from anemia, hemorrhage, and edema due to lymphatic dysfunction (Lim et al., 2012). In addition, conditional loss of Gata2 in lymphatic endothelial cells during development results in dermal lymphatic vessel mispatterning and loss of lymphovenous valves (Frye et al., 2018; Kazenwadel et al., 2015). These elegant mouse studies have established the crucial role of GATA2 not only in hematopoiesis but lymphatic development. Another interesting finding from tissue‐specific knockdown of Gata2 was using an MSC‐specific *Prx1* gene promoter in mice (Hasegawa et al., 2017). Their results suggested that GATA2 regulates cell adhesion and chemotaxis in BM to maintain the BM microenvironment, and that loss of GATA2 in mesenchymal stromal cells (MSC) may contribute to aberrant HSC colony formation (Hasegawa et al., 2017). This finding suggested that GATA2‐associated BM disorders may not solely result from HSC intrinsic processes, but also the BM niche. This is correlated with evidence that GATA2 expression is decreased in BM MSC from patients with aplastic anemia (Xu et al., 2009). In a different context, rescue of hematopoietic deficiency of GATA2 using a *Gata2* yeast artificial chromosome transgene in mice led to the discovery of a contribution to urogenital development (Zhou et al., 1998). These mice suffered from megaureter and hydronephrosis caused by ureters that ended blindly or were aberrantly connected to the seminal vesicle or vas deferens and resulted in perinatal lethality. Interestingly, these findings are consistent with urogenital abnormalities being seen in 5%–12% of G2DS patients (Donadieu et al., 2018; Wlodarski et al., 2016).

Overall, mouse models have contributed greatly to our understanding of the role(s) of GATA2 in the genesis and function of hematopoietic and nonhematopoietic tissues. To date, there are no knockin mouse models that mimic any of the commonly seen human missense variants. Furthermore, few have faithfully modeled the initiation and progression of G2DS phenotypes as seen in human disease (e.g., MDS or AML), possibly due to the short murine lifespan and the different range of challenges and stressors experienced during a human lifetime.

## 2 CONCLUSION

In just one decade, the impact of germline *GATA2* pathogenic variants has been noted around the world, and clinical practice has changed to help patients and their families. This collation of published *GATA2* variants is a powerful resource for helping health professionals in ACMG/AMP classification of identified variants and subsequent clinical management. A better understanding of the types of *GATA2* variants and their impacts on the cellular processes and environmental stressors leading to G2DS phenotypes will enable better surveillance measures, treatments, and ultimately strategies to prevent disease onset.

## CONFLICT OF INTERESTS

The authors declare that there are no conflict of interests.

## AUTHOR CONTRIBUTIONS

Claire C. Homan, Parvathy Venugopal, Peer Arts, Nur H. Shahrin, Christopher N. Hahn, Natasha L. Harvey wrote the manuscript and prepared tables and figures. SF and CNH performed ACMG/AMP classifications. Parvathy Venugopal, David M. Lawrence, James Andrews, Sarah L. King‐Smith, and Christopher N. Hahn configured and transferred data to ClinVar. SF, ALB, NLH, and HSS reviewed and edited the manuscript.

## Supporting information

Supporting information

Supporting information

## Data Availability

The data that supports the findings of this study are available in the supplementary material of this article.
