# Supplementary material for: GATA2 deficiency syndrome: A decade of discovery
Source: Hum Mutat. 2021 Aug 31;42(11):1399–421. doi: 10.1002/humu.24271 (PMC9291163; doi:10.1002/humu.24271)
Supplement: Supplementary file 1 — Supporting information [file HUMU-42-1399-s002.pdf]

Reference list for Supplementary Table 1

Abou Dalle, I., Bannon, S. A., Patel, K. P., Routbort, M. J., Cortes, J. E., Ferrajoli, A., . . . DiNardo, C. D. (2019). Germline Genetic Predisposition to Myeloid Neoplasia From GATA2 Gene Mutations: Lessons Learned From Two Cases. *JCO Precis Oncol, 3*. doi:10.1200/PO.18.00301

Alvarez-Chinchilla, P., Poveda, I., Marco, F. M., Lopez-Fernandez, J. A., Peiro, G., Illan, F., & Guijarro, J. (2017). Vulvar lymphedema and refractory VIN-III heralding GATA2 deficiency syndrome. *Eur J Obstet Gynecol Reprod Biol, 218*, 138-140. doi:10.1016/j.ejogrb.2017.09.016

An, W. B., Liu, C., Wan, Y., Chen, X. Y., Guo, Y., Chen, X. J., . . . Zhu, X. F. (2019). [Clinical and molecular characteristics of GATA2 related pediatric primary myelodysplastic syndrome]. *Zhonghua Xue Ye Xue Za Zhi, 40*(6), 477-483. doi:10.3760/cma.j.issn.0253-2727.2019.06.006

Anderson, R. C., & Anderson, K. E. (1990). Success and failure attributions in smoking cessation among men and women. *AAOHN J, 38*(4), 180-185.

Arts, P., Simons, A., AlZahrani, M. S., Yilmaz, E., AlIdrissi, E., van Aerde, K. J., . . . Hoischen, A. (2019). Exome sequencing in routine diagnostics: a generic test for 254 patients with primary immunodeficiencies. *Genome Med, 11*(1), 38. doi:10.1186/s13073-019-0649-3

Azevedo, L., Nofal, R., Jay, A., Lorenzana, A., Keel, S., Abraham, R. S., . . . Sawaf, H. (2017). Case Report of an Adolescent Male With Unexplained Pancytopenia: GATA2-Associated Bone Marrow Failure and Genetic Testing. *Glob Pediatr Health, 4*, 2333794X17744947. doi:10.1177/2333794X17744947

Ballerie, A., Nimubona, S., Meunier, C., Gutierrez, F. L., Desrues, B., Delaval, P., & Jouneau, S. (2016). Association of pulmonary alveolar proteinosis and fibrosis: patient with GATA2 deficiency. *Eur Respir J, 48*(5), 1510-1514. doi:10.1183/13993003.00252-2016

Bigley, V., & Collin, M. (2011). Dendritic cell, monocyte, B and NK lymphoid deficiency defines the lost lineages of a new GATA-2 dependent myelodysplastic syndrome. *Haematologica, 96*(8), 1081-1083. doi:10.3324/haematol.2011.048355

Biron, C. A., Byron, K. S., & Sullivan, J. L. (1989). Severe herpesvirus infections in an adolescent without natural killer cells. *N Engl J Med, 320*(26), 1731-1735. doi:10.1056/NEJM198906293202605

Blanco, M. L., Torrent, M., Bussaglia, E., Badell, I., & Nomdedeu, J. F. (2020). Many signs, one mutation: Early onset of de novo GATA2 deficiency syndrome. A case report. *Clin Case Rep, 8*(12), 3193-3197. doi:10.1002/ccr3.3304

Bluteau, O., Sebert, M., Leblanc, T., Peffault de Latour, R., Quentin, S., Lainey, E., . . . Soulier, J. (2018). A landscape of germ line mutations in a cohort of inherited bone marrow failure patients. *Blood, 131*(7), 717-732. doi:10.1182/blood-2017-09-806489

Bodor, C., Renneville, A., Smith, M., Charazac, A., Iqbal, S., Etancelin, P., . . . Owen, C. (2012). Germ-line GATA2 p.THR354MET mutation in familial myelodysplastic syndrome with acquired monosomy 7 and ASXL1 mutation demonstrating rapid onset and poor survival. *Haematologica, 97*(6), 890-894. doi:10.3324/haematol.2011.054361

Bogaert, D. J., Laureys, G., Naesens, L., Mazure, D., De Bruyne, M., Hsu, A. P., . . . Kerre, T. (2020). GATA2 deficiency and haematopoietic stem cell transplantation: challenges for the clinical practitioner. *Br J Haematol, 188*(5), 768-773. doi:10.1111/bjh.16247

Brambila-Tapia, A. J. L., Garcia-Ortiz, J. E., Brouillard, P., Nguyen, H. L., Vikkula, M., Rios-Gonzalez, B. E., . . . Arnaud-Lopez, L. (2017). GATA2 null mutation associated with incomplete penetrance in a family with Emberger syndrome. *Hematology, 22*(8), 467-471. doi:10.1080/10245332.2017.1294551

Burak, N., Jan, N., Kessler, J., Oei, E., Patel, P., & Feldman, S. (2021). Diagnosis of GATA2 Deficiency in a Young Woman with Hemophagocytic Lymphohistiocytosis Triggered by Acute Systemic Cytomegalovirus Infection. *Am J Case Rep, 22*, e927087. doi:10.12659/AJCR.927087

Burroughs, L. M., Shimamura, A., Talano, J. A., Domm, J. A., Baker, K. K., Delaney, C., . . . Woolfrey, A. E. (2017). Allogeneic Hematopoietic Cell Transplantation Using Treosulfan-Based

Conditioning for Treatment of Marrow Failure Disorders. *Biol Blood Marrow Transplant, 23*(10), 1669-1677. doi:10.1016/j.bbmt.2017.06.002

Callier, P., Faivre, L., Marle, N., Thauvin-Robinet, C., Guy, J., Mosca, A. L., . . . Mugneret, F. (2009). Detection of an interstitial 3q21.1-q21.3 deletion in a child with multiple congenital abnormalities, mental retardation, pancytopenia, and myelodysplasia. *Am J Med Genet A, 149A*(6), 1323-1326. doi:10.1002/ajmg.a.32857

Camargo, J. F., Lobo, S. A., Hsu, A. P., Zerbe, C. S., Wormser, G. P., & Holland, S. M. (2013). MonoMAC syndrome in a patient with a GATA2 mutation: case report and review of the literature. *Clin Infect Dis, 57*(5), 697-699. doi:10.1093/cid/cit368

Celton, M., Forest, A., Gosse, G., Lemieux, S., Hebert, J., Sauvageau, G., & Wilhelm, B. T. (2014). Epigenetic regulation of GATA2 and its impact on normal karyotype acute myeloid leukemia. *Leukemia, 28*(8), 1617-1626. doi:10.1038/leu.2014.67

Churpek, J. E., Pyrtel, K., Kanchi, K. L., Shao, J., Koboldt, D., Miller, C. A., . . . Graubert, T. A. (2015). Genomic analysis of germ line and somatic variants in familial myelodysplasia/acute myeloid leukemia. *Blood, 126*(22), 2484-2490. doi:10.1182/blood-2015-04-641100

Cortes-Lavaud, X., Landecho, M. F., Maicas, M., Urquiza, L., Merino, J., Moreno-Miralles, I., & Odero, M. D. (2015). GATA2 germline mutations impair GATA2 transcription, causing haploinsufficiency: functional analysis of the p.Arg396Gln mutation. *J Immunol, 194*(5), 2190-2198. doi:10.4049/jimmunol.1401868

Cuellar-Rodriguez, J., Gea-Banacloche, J., Freeman, A. F., Hsu, A. P., Zerbe, C. S., Calvo, K. R., . . . Hickstein, D. D. (2011). Successful allogeneic hematopoietic stem cell transplantation for GATA2 deficiency. *Blood, 118*(13), 3715-3720. doi:10.1182/blood-2011-06-365049

Damian, L., Sauvetre, G., Marguet, F., Verdalle-Cazes, M., Battistella, M., & Boutboul, D. (2018). Pseudo-Sarcoidosis Revealing MonoMAC Syndrome. *J Clin Immunol, 38*(7), 739-741. doi:10.1007/s10875-018-0551-6

Delgado-Marquez, A. M., Zarco, C., Ruiz, R., Simarro, A., & Vanaclocha, F. (2016). Severe disseminated primary herpes simplex infection as skin manifestation of GATA2 deficiency. *J Eur Acad Dermatol Venereol, 30*(7), 1248-1250. doi:10.1111/jdv.13183

Dickinson, R. E., Griffin, H., Bigley, V., Reynard, L. N., Hussain, R., Haniffa, M., . . . Collin, M. (2011). Exome sequencing identifies GATA-2 mutation as the cause of dendritic cell, monocyte, B and NK lymphoid deficiency. *Blood, 118*(10), 2656-2658. doi:10.1182/blood-2011-06-360313

Dickinson, R. E., Milne, P., Jardine, L., Zandi, S., Swierczek, S. I., McGovern, N., . . . Collin, M. (2014). The evolution of cellular deficiency in GATA2 mutation. *Blood, 123*(6), 863-874. doi:10.1182/blood-2013-07-517151

Ding, L. W., Ikezoe, T., Tan, K. T., Mori, M., Mayakonda, A., Chien, W., . . . Koeffler, H. P. (2017). Mutational profiling of a MonoMAC syndrome family with GATA2 deficiency. *Leukemia, 31*(1), 244-245. doi:10.1038/leu.2016.256

Donadieu, J., Lamant, M., Fieschi, C., de Fontbrune, F. S., Caye, A., Ouachee, M., . . . French, G. s. g. (2018). Natural history of GATA2 deficiency in a survey of 79 French and Belgian patients. *Haematologica, 103*(8), 1278-1287. doi:10.3324/haematol.2017.181909

Dorn, J. M., Patnaik, M. S., Van Hee, M., Smith, M. J., Lagerstedt, S. A., Newman, C. C., . . . Abraham, R. S. (2017). WILD syndrome is GATA2 deficiency: A novel deletion in the GATA2 gene. *J Allergy Clin Immunol Pract, 5*(4), 1149-1152 e1141. doi:10.1016/j.jaip.2017.02.010

Drazer, M. W., Kadri, S., Sukhanova, M., Patil, S. A., West, A. H., Feurstein, S., . . . Godley, L. A. (2018). Prognostic tumor sequencing panels frequently identify germ line variants associated with hereditary hematopoietic malignancies. *Blood Adv, 2*(2), 146-150. doi:10.1182/bloodadvances.2017013037

Ellingford, J. M., Telford, N., Urquhart, J., Will, A. M., Bonney, D., Adams, B., . . . Meyer, S. (2021). High penetrance of myeloid neoplasia with diverse clinical and cytogenetic features in three siblings with a familial GATA2 deficiency. *Cancer Genet, 256-257*, 77-80. doi:10.1016/j.cancergen.2021.04.002

Esparza, O., Xavier, A. C., Atkinson, T. P., Hill, B. C., & Whelan, K. (2019). A unique phenotype of T-cell acute lymphoblastic leukemia in a patient with GATA2 haploinsufficiency. *Pediatr Blood Cancer, 66*(6), e27649. doi:10.1002/pbc.27649

Feurstein, S., Churpek, J. E., Walsh, T., Keel, S., Hakkarainen, M., Schroeder, T., . . . Godley, L. A. (2021). Germline variants drive myelodysplastic syndrome in young adults. *Leukemia*. doi:10.1038/s41375-021-01137-0

Fox, L. C., Tan, M., Brown, A. L., Arts, P., Thompson, E., Ryland, G. L., . . . Blombery, P. (2020). A synonymous GATA2 variant underlying familial myeloid malignancy with striking intrafamilial phenotypic variability. *Br J Haematol, 190*(5), e297-e301. doi:10.1111/bjh.16819

Fox, T. A., Chakraverty, R., Burns, S., Carpenter, B., Thomson, K., Lowe, D., . . . Morris, E. (2018). Successful outcome following allogeneic hematopoietic stem cell transplantation in adults with primary immunodeficiency. *Blood, 131*(8), 917-931. doi:10.1182/blood-2017-09-807487

Fujiwara, T., Fukuhara, N., Funayama, R., Nariai, N., Kamata, M., Nagashima, T., . . . Harigae, H. (2014). Identification of acquired mutations by whole-genome sequencing in GATA-2 deficiency evolving into myelodysplasia and acute leukemia. *Ann Hematol, 93*(9), 1515-1522. doi:10.1007/s00277-014-2090-4

Ganapathi, K. A., Townsley, D. M., Hsu, A. P., Arthur, D. C., Zerbe, C. S., Cuellar-Rodriguez, J., . . . Calvo, K. R. (2015). GATA2 deficiency-associated bone marrow disorder differs from idiopathic aplastic anemia. *Blood, 125*(1), 56-70. doi:10.1182/blood-2014-06-580340

Gao, J., Gentzler, R. D., Timms, A. E., Horwitz, M. S., Frankfurt, O., Altman, J. K., & Peterson, L. C. (2014). Heritable GATA2 mutations associated with familial AML-MDS: a case report and review of literature. *J Hematol Oncol, 7*, 36. doi:10.1186/1756-8722-7-36

Gonzalez-Lara, M. F., Wisniowski-Yanez, A., Perez-Patrigeon, S., Hsu, A. P., Holland, S. M., & Cuellar-Rodriguez, J. M. (2017). Pneumocystis jiroveci pneumonia and GATA2 deficiency: Expanding the spectrum of the disease. *J Infect, 74*(4), 425-427. doi:10.1016/j.jinf.2017.01.005

Griese, M., Zarbock, R., Costabel, U., Hildebrandt, J., Theegarten, D., Albert, M., . . . Bonella, F. (2015). GATA2 deficiency in children and adults with severe pulmonary alveolar proteinosis and hematologic disorders. *BMC Pulm Med, 15*, 87. doi:10.1186/s12890-015-0083-2

Guidugli, L., Johnson, A. K., Alkorta-Aranburu, G., Nelakuditi, V., Arndt, K., Churpek, J. E., . . . Li, Z. (2017). Clinical utility of gene panel-based testing for hereditary myelodysplastic syndrome/acute leukemia predisposition syndromes. *Leukemia, 31*(5), 1226-1229. doi:10.1038/leu.2017.28

Hahn, C. N., Brautigan, P. J., Chong, C. E., Janssan, A., Venugopal, P., Lee, Y., . . . Scott, H. S. (2015). Characterisation of a compound in-cis GATA2 germline mutation in a pedigree presenting with myelodysplastic syndrome/acute myeloid leukemia with concurrent thrombocytopenia. *Leukemia, 29*(8), 1795-1797. doi:10.1038/leu.2015.40

Hahn, C. N., Chong, C. E., Carmichael, C. L., Wilkins, E. J., Brautigan, P. J., Li, X. C., . . . Scott, H. S. (2011). Heritable GATA2 mutations associated with familial myelodysplastic syndrome and acute myeloid leukemia. *Nat Genet, 43*(10), 1012-1017. doi:10.1038/ng.913

Hofmann, I., Avagyan, S., Stetson, A., Guo, D., Al-Sayegh, H., London, W. B., & Lehmann, L. (2020). Comparison of Outcomes of Myeloablative Allogeneic Stem Cell Transplantation for Pediatric Patients with Bone Marrow Failure, Myelodysplastic Syndrome and Acute Myeloid Leukemia with and without Germline GATA2 Mutations. *Biol Blood Marrow Transplant, 26*(6), 1124-1130. doi:10.1016/j.bbmt.2020.02.015

Holme, H., Hossain, U., Kirwan, M., Walne, A., Vulliamy, T., & Dokal, I. (2012). Marked genetic heterogeneity in familial myelodysplasia/acute myeloid leukaemia. *Br J Haematol, 158*(2), 242-248. doi:10.1111/j.1365-2141.2012.09136.x

Horwitz, M., Sabath, D. E., Smithson, W. A., & Radich, J. (1996). A family inheriting different subtypes of acute myelogenous leukemia. *Am J Hematol, 52*(4), 295-304. doi:10.1002/(SICI)1096-8652(199608)52:4<295::AID-AJH9>3.0.CO;2-N

Hsu, A. P., Johnson, K. D., Falcone, E. L., Sanalkumar, R., Sanchez, L., Hickstein, D. D., . . . Holland, S. M. (2013). GATA2 haploinsufficiency caused by mutations in a conserved intronic element leads to MonoMAC syndrome. *Blood, 121*(19), 3830-3837, S3831-3837. doi:10.1182/blood-2012-08-452763

Hsu, A. P., Sampaio, E. P., Khan, J., Calvo, K. R., Lemieux, J. E., Patel, S. Y., . . . Holland, S. M. (2011). Mutations in GATA2 are associated with the autosomal dominant and sporadic monocytopenia and mycobacterial infection (MonoMAC) syndrome. *Blood, 118*(10), 2653-2655. doi:10.1182/blood-2011-05-356352

Ishida, H., Imai, K., Honma, K., Tamura, S., Imamura, T., Ito, M., & Nonoyama, S. (2012). GATA-2 anomaly and clinical phenotype of a sporadic case of lymphedema, dendritic cell, monocyte, B- and NK-cell (DCML) deficiency, and myelodysplasia. *Eur J Pediatr, 171*(8), 1273-1276. doi:10.1007/s00431-012-1715-7

Jensen, M. L. N., Mathiasen, V. D., Ifversen, M., & Nielsen, J. S. A. (2020). Severe influenza in a paediatric patient with GATA2 deficiency and Emberger syndrome. *BMJ Case Rep, 13*(12). doi:10.1136/bcr-2020-236521

Johnson, K. D., Hsu, A. P., Ryu, M. J., Wang, J., Gao, X., Boyer, M. E., . . . Bresnick, E. H. (2012). Cis-element mutated in GATA2-dependent immunodeficiency governs hematopoiesis and vascular integrity. *J Clin Invest, 122*(10), 3692-3704. doi:10.1172/JCI61623

Kaur, J., Catovsky, D., Valdimarsson, H., Jensson, O., & Spiers, A. S. (1972). Familial acute myeloid leukaemia with acquired Pelger-Huet anomaly and aneuploidy of C group. *Br Med J, 4*(5836), 327-331. doi:10.1136/bmj.4.5836.327

Kazenwadel, J., Secker, G. A., Liu, Y. J., Rosenfeld, J. A., Wildin, R. S., Cuellar-Rodriguez, J., . . . Harvey, N. L. (2012). Loss-of-function germline GATA2 mutations in patients with MDS/AML or MonoMAC syndrome and primary lymphedema reveal a key role for GATA2 in the lymphatic vasculature. *Blood, 119*(5), 1283-1291. doi:10.1182/blood-2011-08-374363

Koegel, A. K., Hofmann, I., Moffitt, K., Degar, B., Duncan, C., & Tubman, V. N. (2016). Acute lymphoblastic leukemia in a patient with MonoMAC syndrome/GATA2 haploinsufficiency. *Pediatr Blood Cancer, 63*(10), 1844-1847. doi:10.1002/pbc.26084

Kozyra, E. J., Pastor, V. B., Lefkopoulos, S., Sahoo, S. S., Busch, H., Voss, R. K., . . . European Working Group of, M. D. S. i. C. (2020). Synonymous GATA2 mutations result in selective loss of mutated RNA and are common in patients with GATA2 deficiency. *Leukemia, 34*(10), 2673-2687. doi:10.1038/s41375-020-0899-5

Kurata, T., Shigemura, T., Muramatsu, H., Okuno, Y., & Nakazawa, Y. (2017). A case of GATA2-related myelodysplastic syndrome with unbalanced translocation der(1;7)(q10;p10). *Pediatr Blood Cancer, 64*(8). doi:10.1002/pbc.26419

Kuriyama, Y., Hattori, M., Mitsui, T., Nakano, H., Oikawa, D., Tokunaga, F., . . . Shimizu, A. (2018). Generalized verrucosis caused by various human papillomaviruses in a patient with GATA2 deficiency. *J Dermatol, 45*(5), e108-e109. doi:10.1111/1346-8138.14149

Lee, H., Deignan, J. L., Dorrani, N., Strom, S. P., Kantarci, S., Quintero-Rivera, F., . . . Nelson, S. F. (2014). Clinical exome sequencing for genetic identification of rare Mendelian disorders. *JAMA, 312*(18), 1880-1887. doi:10.1001/jama.2014.14604

Lubking, A., Vosberg, S., Konstandin, N. P., Dufour, A., Graf, A., Krebs, S., . . . Cammenga, J. (2015). Young woman with mild bone marrow dysplasia, GATA2 and ASXL1 mutation treated with allogeneic hematopoietic stem cell transplantation. *Leuk Res Rep, 4*(2), 72-75. doi:10.1016/j.lrr.2015.10.001

Mace, E. M., Hsu, A. P., Monaco-Shawver, L., Makedonas, G., Rosen, J. B., Dropulic, L., . . . Orange, J. S. (2013). Mutations in GATA2 cause human NK cell deficiency with specific loss of the CD56(bright) subset. *Blood, 121*(14), 2669-2677. doi:10.1182/blood-2012-09-453969

Maciejewski-Duval, A., Meuris, F., Bignon, A., Aknin, M. L., Balabanian, K., Faivre, L., . . . Bachelerie, F. (2016). Altered chemotactic response to CXCL12 in patients carrying GATA2 mutations. *J Leukoc Biol, 99*(6), 1065-1076. doi:10.1189/jlb.5MA0815-388R

Mallhi, K., Dix, D. B., Niederhoffer, K. Y., Armstrong, L., & Rozmus, J. (2016). Successful umbilical cord blood hematopoietic stem cell transplantation in pediatric patients with MDS/AML associated with underlying GATA2 mutations: two case reports and review of literature. *Pediatr Transplant, 20*(7), 1004-1007. doi:10.1111/petr.12764

Mangaonkar, A. A., Ferrer, A., Pinto, E. V. F., Cousin, M. A., Kuisle, R. J., Gangat, N., . . . Patnaik, M. M. (2019). Clinical Applications and Utility of a Precision Medicine Approach for Patients With Unexplained Cytopenias. *Mayo Clin Proc, 94*(9), 1753-1768. doi:10.1016/j.mayocp.2019.04.007

Mardahl, M., Jorgensen, S. E., Schneider, A., Raaschou-Jensen, K., Holm, M., Veirum, J., . . . Mogensen, T. H. (2019). Impaired immune responses to herpesviruses and microbial ligands in patients with MonoMAC. *Br J Haematol, 186*(3), 471-476. doi:10.1111/bjh.15947

McReynolds, L. J., Yang, Y., Yuen Wong, H., Tang, J., Zhang, Y., Mule, M. P., . . . Hourigan, C. S. (2019). MDS-associated mutations in germline GATA2 mutated patients with hematologic manifestations. *Leuk Res, 76*, 70-75. doi:10.1016/j.leukres.2018.11.013

McReynolds, L. J., Zhang, Y., Yang, Y., Tang, J., Mule, M., Hsu, A. P., . . . Hourigan, C. S. (2019). Rapid progression to AML in a patient with germline GATA2 mutation and acquired NRAS Q61K mutation. *Leuk Res Rep, 12*, 100176. doi:10.1016/j.lrr.2019.100176

Mendes-de-Almeida, D. P., Andrade, F. G., Borges, G., Dos Santos-Bueno, F. V., Vieira, I. F., da Rocha, L., . . . Pombo-de-Oliveira, M. S. (2019). GATA2 mutation in long stand Mycobacterium kansasii infection, myelodysplasia and MonoMAC syndrome: a case-report. *BMC Med Genet, 20*(1), 64. doi:10.1186/s12881-019-0799-6

Mendola, A., Schlogel, M. J., Ghalamkarpour, A., Irrthum, A., Nguyen, H. L., Fastre, E., . . . Lymphedema Research, G. (2013). Mutations in the VEGFR3 signaling pathway explain 36% of familial lymphedema. *Mol Syndromol, 4*(6), 257-266. doi:10.1159/000354097

Metzeler, K. H., Herold, T., Rothenberg-Thurley, M., Amler, S., Sauerland, M. C., Gorlich, D., . . . Group, A. S. (2016). Spectrum and prognostic relevance of driver gene mutations in acute myeloid leukemia. *Blood, 128*(5), 686-698. doi:10.1182/blood-2016-01-693879

Michelini, S., Cardone, M., Haag, M., Agga, O., Bruson, A., Maltese, P. E., . . . Bertelli, M. (2016). A Rare Case of Emberger Syndrome Caused By a De Novo Mutation in the GATA2 Gene. *Lymphology, 49*(1), 15-20.

Mir, M. A., Kochuparambil, S. T., Abraham, R. S., Rodriguez, V., Howard, M., Hsu, A. P., . . . Patnaik, M. M. (2015). Spectrum of myeloid neoplasms and immune deficiency associated with germline GATA2 mutations. *Cancer Med, 4*(4), 490-499. doi:10.1002/cam4.384

Mojica, A. M., & Elizalde, A. (2019). GATA2 Deficiency in a Pediatric Patient. *J Allergy Clin Immunol Pract, 7*(6), 2021-2022. doi:10.1016/j.jaip.2019.02.024

Monif, M., Huq, A., Chee, L., & Kilpatrick, T. (2018). MonoMac syndrome with associated neurological deficits and longitudinally extensive cord lesion. *BMJ Case Rep, 2018*. doi:10.1136/bcr-2017-222872

Mutsaers, P. G., van de Loosdrecht, A. A., Tawana, K., Bodor, C., Fitzgibbon, J., & Menko, F. H. (2013). Highly variable clinical manifestations in a large family with a novel GATA2 mutation. *Leukemia, 27*(11), 2247-2248. doi:10.1038/leu.2013.105

Nakazawa, H., Yamaguchi, T., Sakai, H., Maruyama, M., Kawakami, T., Kawakami, F., . . . Ishida, F. (2021). A novel germline GATA2 frameshift mutation with a premature stop codon in a family with congenital sensory hearing loss and myelodysplastic syndrome. *Int J Hematol*. doi:10.1007/s12185-021-03130-w

Nguyen, J., Alexander, T., Jiang, H., Hill, N., Abdullaev, Z., Pack, S. D., . . . Brownell, I. (2018). Melanoma in patients with GATA2 deficiency. *Pigment Cell Melanoma Res, 31*(2), 337-340. doi:10.1111/pcmr.12671

Novakova, M., Zaliova, M., Sukova, M., Wlodarski, M., Janda, A., Fronkova, E., . . . Mejstrikova, E. (2016). Loss of B cells and their precursors is the most constant feature of GATA-2 deficiency

in childhood myelodysplastic syndrome. *Haematologica, 101*(6), 707-716. doi:10.3324/haematol.2015.137711

Oleaga-Quintas, C., de Oliveira-Junior, E. B., Rosain, J., Rapaport, F., Deswarte, C., Guerin, A., . . . Bustamante, J. (2021). Inherited GATA2 Deficiency Is Dominant by Haploinsufficiency and Displays Incomplete Clinical Penetrance. *J Clin Immunol, 41*(3), 639-657. doi:10.1007/s10875-020-00930-3

Ostergaard, P., Simpson, M. A., Connell, F. C., Steward, C. G., Brice, G., Woollard, W. J., . . . Mansour, S. (2011). Mutations in GATA2 cause primary lymphedema associated with a predisposition to acute myeloid leukemia (Emberger syndrome). *Nat Genet, 43*(10), 929-931. doi:10.1038/ng.923

Parta, M., Cuellar-Rodriguez, J., Freeman, A. F., Gea-Banacloche, J., Holland, S. M., & Hickstein, D. D. (2017). Resolution of Multifocal Epstein-Barr Virus-Related Smooth Muscle Tumor in a Patient with GATA2 Deficiency Following Hematopoietic Stem Cell Transplantation. *J Clin Immunol, 37*(1), 61-66. doi:10.1007/s10875-016-0360-8

Pasquet, M., Bellanne-Chantelot, C., Tavitian, S., Prade, N., Beaupain, B., Larochelle, O., . . . Delabesse, E. (2013). High frequency of GATA2 mutations in patients with mild chronic neutropenia evolving to MonoMac syndrome, myelodysplasia, and acute myeloid leukemia. *Blood, 121*(5), 822-829. doi:10.1182/blood-2012-08-447367

Polat, A., Dinulescu, M., Fraitag, S., Nimubona, S., Toutain, F., Jouneau, S., . . . Dupuy, A. (2018). Skin manifestations among GATA2-deficient patients. *Br J Dermatol, 178*(3), 781-785. doi:10.1111/bjd.15548

Prader, S., Felber, M., Volkmer, B., Truck, J., Schwieger-Briel, A., Theiler, M., . . . Pachlopnik Schmid, J. (2018). Life-Threatening Primary Varicella Zoster Virus Infection With Hemophagocytic Lymphohistiocytosis-Like Disease in GATA2 Haploinsufficiency Accompanied by Expansion of Double Negative T-Lymphocytes. *Front Immunol, 9*, 2766. doi:10.3389/fimmu.2018.02766

Ramzan, M., Lowry, J., Courtney, S., Krueger, J., Schechter Finkelstein, T., & Ali, M. (2017). Successful Myeloablative Matched Unrelated Donor Hematopoietic Stem Cell Transplantation in a Young Girl With GATA2 Deficiency and Emberger Syndrome. *J Pediatr Hematol Oncol, 39*(3), 230-232. doi:10.1097/MPH.0000000000000737

Rastogi, N., Abraham, R. S., Chadha, R., Thakkar, D., Kohli, S., Nivargi, S., & Prakash Yadav, S. (2018). Successful Nonmyeloablative Allogeneic Stem Cell Transplant in a Child With Emberger Syndrome and GATA2 Mutation. *J Pediatr Hematol Oncol, 40*(6), e383-e388. doi:10.1097/MPH.0000000000000995

Rosa, J. S., Kappagoda, S., Hsu, A. P., Davis, J., Holland, S. M., & Liu, A. Y. (2019). West Nile virus encephalitis in GATA2 deficiency. *Allergy Asthma Clin Immunol, 15*, 5. doi:10.1186/s13223-019-0321-x

Ruiz-Garcia, R., Rodriguez-Vigil, C., Marco, F. M., Gallego-Bustos, F., Castro-Panete, M. J., Diez-Alonso, L., . . . Allende, L. M. (2017). Acquired Senescent T-Cell Phenotype Correlates with Clinical Severity in GATA Binding Protein 2-Deficient Patients. *Front Immunol, 8*, 802. doi:10.3389/fimmu.2017.00802

Saida, S., Umeda, K., Yasumi, T., Matsumoto, A., Kato, I., Hiramatsu, H., . . . Adachi, S. (2016). Successful reduced-intensity stem cell transplantation for GATA2 deficiency before progression of advanced MDS. *Pediatr Transplant, 20*(2), 333-336. doi:10.1111/petr.12667

Sakata, N., Okano, M., Masako, R., Tanaka, A., Yamashita, Y., Karasuno, T., . . . Sugimoto, K. (2021). Donor-derived myelodysplastic syndrome after allogeneic stem cell transplantation in a family with germline GATA2 mutation. *Int J Hematol, 113*(2), 290-296. doi:10.1007/s12185-020-02980-0

Sanyi, A., Jaye, D. L., Rosand, C. B., Box, A., Shanmuganathan, C., & Waller, E. K. (2018). Diagnosis of GATA2 haplo-insufficiency in a young woman prompted by pancytopenia with deficiencies of B-cell and dendritic cell development. *Biomark Res, 6*, 13. doi:10.1186/s40364-018-0127-x

Schlums, H., Jung, M., Han, H., Theorell, J., Bigley, V., Chiang, S. C., . . . Bryceson, Y. T. (2017). Adaptive NK cells can persist in patients with GATA2 mutation depleted of stem and progenitor cells. *Blood, 129*(14), 1927-1939. doi:10.1182/blood-2016-08-734236

Schwartz, J. R., Ma, J., Lamprecht, T., Walsh, M., Wang, S., Bryant, V., . . . Klco, J. M. (2017). The genomic landscape of pediatric myelodysplastic syndromes. *Nat Commun, 8*(1), 1557. doi:10.1038/s41467-017-01590-5

Seo, S. K., Kim, K. Y., Han, S. A., Yoon, J. S., Shin, S. Y., Sohn, S. K., & Moon, J. H. (2016). First Korean case of Emberger syndrome (primary lymphedema with myelodysplasia) with a novel GATA2 gene mutation. *Korean J Intern Med, 31*(1), 188-190. doi:10.3904/kjim.2016.31.1.188

Shah, N. N., Parta, M., Baird, K., Rafei, H., Cole, K., Holland, S. M., & Hickstein, D. D. (2017). Monozygotic twins with GATA2 deficiency: same haploidentical-related donor, different severity of GvHD. *Bone Marrow Transplant, 52*(11), 1580-1582. doi:10.1038/bmt.2017.180

Simon, A. J., Golan, A. C., Lev, A., Stauber, T., Barel, O., Somekh, I., . . . Somech, R. (2020). Whole exome sequencing (WES) approach for diagnosing primary immunodeficiencies (PIDs) in a highly consanguineous community. *Clin Immunol, 214*, 108376. doi:10.1016/j.clim.2020.108376

Simonis, A., Fux, M., Nair, G., Mueller, N. J., Haralambieva, E., Pabst, T., . . . Muller, A. M. S. (2018). Allogeneic hematopoietic cell transplantation in patients with GATA2 deficiency-a case report and comprehensive review of the literature. *Ann Hematol, 97*(10), 1961-1973. doi:10.1007/s00277-018-3388-4

Sologuren, I., Martinez-Saavedra, M. T., Sole-Violan, J., de Borges de Oliveira, E., Jr., Betancor, E., Casas, I., . . . Rodriguez-Gallego, C. (2018). Lethal Influenza in Two Related Adults with Inherited GATA2 Deficiency. *J Clin Immunol, 38*(4), 513-526. doi:10.1007/s10875-018-0512-0

Spinner, M. A., Sanchez, L. A., Hsu, A. P., Shaw, P. A., Zerbe, C. S., Calvo, K. R., . . . Holland, S. M. (2014). GATA2 deficiency: a protean disorder of hematopoiesis, lymphatics, and immunity. *Blood, 123*(6), 809-821. doi:10.1182/blood-2013-07-515528

Stieglitz, E., Liu, Y. L., Emanuel, P. D., Castleberry, R. P., Cooper, T. M., Shannon, K. M., & Loh, M. L. (2014). Mutations in GATA2 are rare in juvenile myelomonocytic leukemia. *Blood, 123*(9), 1426-1427. doi:10.1182/blood-2013-11-531079

Stray-Pedersen, A., Sorte, H. S., Samarakoon, P., Gambin, T., Chinn, I. K., Coban Akdemir, Z. H., . . . Lupski, J. R. (2017). Primary immunodeficiency diseases: Genomic approaches delineate heterogeneous Mendelian disorders. *J Allergy Clin Immunol, 139*(1), 232-245. doi:10.1016/j.jaci.2016.05.042

Suzuki, T., Takaya, S., Kunimatsu, J., Kutsuna, S., Hayakawa, K., Shibata, H., . . . Ohmagari, N. (2020). GATA2 mutation underlies hemophagocytic lymphohistiocytosis in an adult with primary cytomegalovirus infection. *J Infect Chemother, 26*(2), 252-256. doi:10.1016/j.jiac.2019.07.002

Svobodova, T., Mejstrikova, E., Salzer, U., Sukova, M., Hubacek, P., Matej, R., . . . Janda, A. (2015). Diffuse parenchymal lung disease as first clinical manifestation of GATA-2 deficiency in childhood. *BMC Pulm Med, 15*, 8. doi:10.1186/s12890-015-0006-2

van der Horst, R., Hastreiter, A. R., Levitsky, S., Fisher, E. A., DuBrow, I. W., & Weinberg, M. (1979). Interrupted aortic arch operation in the first week of life: hemodynamic and angiographic evaluation one year later. *Ann Thorac Surg, 27*(2), 112-120. doi:10.1016/s0003-4975(10)63250-x

Vila, A., Dapas, J. I., Rivero, C. V., Bocanegra, F., Furnari, R. F., Hsu, A. P., & Holland, S. M. (2017). Multiple Opportunistic Infections in a Woman with GATA2 Mutation. *Int J Infect Dis, 54*, 89-91. doi:10.1016/j.ijid.2016.11.408

Vinh, D. C., Palma, L., Storring, J., & Foulkes, W. D. (2018). GATA2 Deficiency Due to de Novo Complete Monoallelic Deletion in an Adolescent With Myelodysplasia. *J Pediatr Hematol Oncol, 40*(4), e225-e228. doi:10.1097/MPH.0000000000001136

Vinh, D. C., Patel, S. Y., Uzel, G., Anderson, V. L., Freeman, A. F., Olivier, K. N., . . . Holland, S. M. (2010). Autosomal dominant and sporadic monocytopenia with susceptibility to mycobacteria, fungi, papillomaviruses, and myelodysplasia. *Blood, 115*(8), 1519-1529. doi:10.1182/blood-2009-03-208629

Walter, M. J., Shen, D., Shao, J., Ding, L., White, B. S., Kandoth, C., . . . Graubert, T. A. (2013). Clonal diversity of recurrently mutated genes in myelodysplastic syndromes. *Leukemia, 27*(6), 1275-1282. doi:10.1038/leu.2013.58

Wang, X., Muramatsu, H., Okuno, Y., Sakaguchi, H., Yoshida, K., Kawashima, N., . . . Kojima, S. (2015). GATA2 and secondary mutations in familial myelodysplastic syndromes and pediatric myeloid malignancies. *Haematologica, 100*(10), e398-401. doi:10.3324/haematol.2015.127092

Wehr, C., Grotius, K., Casadei, S., Bleckmann, D., Bode, S. F. N., Frye, B. C., . . . Salzer, U. (2018). A novel disease-causing synonymous exonic mutation in GATA2 affecting RNA splicing. *Blood, 132*(11), 1211-1215. doi:10.1182/blood-2018-03-837336

West, E. S., Kingsbery, M. Y., Mintz, E. M., Hsu, A. P., Holland, S. M., Rady, P. L., . . . Grossman, M. E. (2014). Generalized verrucosis in a patient with GATA2 deficiency. *Br J Dermatol, 170*(5), 1182-1186. doi:10.1111/bjd.12794

West, R. R., Hsu, A. P., Holland, S. M., Cuellar-Rodriguez, J., & Hickstein, D. D. (2014). Acquired ASXL1 mutations are common in patients with inherited GATA2 mutations and correlate with myeloid transformation. *Haematologica, 99*(2), 276-281. doi:10.3324/haematol.2013.090217

Wlodarski, M. W., Hirabayashi, S., Pastor, V., Stary, J., Hasle, H., Masetti, R., . . . Ewog, M. D. S. (2016). Prevalence, clinical characteristics, and prognosis of GATA2-related myelodysplastic syndromes in children and adolescents. *Blood, 127*(11), 1387-1397; quiz 1518. doi:10.1182/blood-2015-09-669937

Zhang, M. Y., Keel, S. B., Walsh, T., Lee, M. K., Gulsuner, S., Watts, A. C., . . . Shimamura, A. (2015). Genomic analysis of bone marrow failure and myelodysplastic syndromes reveals phenotypic and diagnostic complexity. *Haematologica, 100*(1), 42-48. doi:10.3324/haematol.2014.113456
